# Supplementary material for: New 8-C-p-Hydroxylbenzylflavonol Glycosides from Pumpkin (Cucurbita moschata Duch.) Tendril and Their Osteoclast Differentiation Inhibitory Activities
Source: Molecules. 2020 Apr 29;25(9):2077. doi: 10.3390/molecules25092077 (PMC7248980; doi:10.3390/molecules25092077)
Supplement: Supplementary file 1 [file molecules-25-02077-s001.pdf]

Supplemental data

Table S1. ^{1}H NMR (500 MHz) data of **1–6** in CD_3OD ········ 1

Table S2. ^{13}C NMR (125 MHz) data of **1–6** in CD_3OD ········ 1

Figure S1. ^{1}H NMR (500 MHz, CD_3OD) spectrum of compound **1**. ········ 2

Figure S2. ^{13}C NMR (125 MHz, CD_3OD) spectrum of compound **1**. ········ 2

Figure S3. HSQC spectrum of compound **1**. ········ 3

Figure S4. ^{1}H-^{1}H COSY spectrum of compound **1**. ········ 3

Figure S5. HMBC spectrum of compound **1**. ········ 4

Figure S6. ESI-MS spectrum of compound **1**. ········ 4

Figure S7. HRESI-MS of compound **1**. ········ 5

Figure S8. ^{1}H NMR (500 MHz, CD_3OD) spectrum of compound **2**. ········ 5

Figure S9. ^{13}C NMR (125 MHz, CD_3OD) spectrum of compound **2**. ········ 6

Figure S10. HSQC spectrum of compound **2**. ········ 6

Figure S11. ^{1}H-^{1}H COSY spectrum of compound **2**. ········ 7

Figure S12. HMBC spectrum of compound **2**. ········ 7

Figure S13. ESI-MS spectrum of compound **2**. ········ 8

Figure S14. HRESI-MS of compound **2**. ········ 8

Figure S15. ^{1}H NMR (500 MHz, CD_3OD) spectrum of compound **3**. ········ 9

Figure S16. ^{13}C NMR (125 MHz, CD_3OD) spectrum of compound **3**. ········ 9

Figure S17. HSQC spectrum of compound **3**. ········ 10

Figure S18. ^{1}H-^{1}H COSY spectrum of compound **3**. ········ 10

Figure S19. HMBC spectrum of compound **3**. ········ 11

Figure S20. ESI-MS spectrum of compound **3**. ········ 11

Figure S21. HRESI-MS of compound **3**. ········ 12

Figure S22. ^{1}H NMR (500 MHz, CD_3OD) spectrum of compound **4**. ········ 12

Figure S23. ^{13}C NMR (125 MHz, CD_3OD) spectrum of compound **4**. ········ 13

Figure S24. HSQC spectrum of compound **4**. ········ 13

Figure S25. ^{1}H-^{1}H COSY spectrum of compound **4**. 14
Figure S26. HMBC spectrum of compound **4**. 14
Figure S27. ^{1}H NMR (500 MHz, DMSO-d_6) spectrum of compound **4**. 15
Figure S28. ^{13}C NMR (125 MHz, DMSO-d_6) spectrum of compound **4**. 15
Figure S29. HSQC spectrum of compound **4**. 16
Figure S30. HMBC spectrum of compound **4**. 16
Figure S31. ROESY spectrum of compound **4**. 17
Figure S32. ESI-MS spectrum of compound **4**. 17
Figure S33. HRESI-MS of compound **4**. 18
Figure S34. ^{1}H NMR (500 MHz, CD_3OD) spectrum of compounds **5** and **6**. 18
Figure S35. ^{13}C NMR (125 MHz, CD_3OD) spectrum of compounds **5** and **6**. 19
Figure S36. HSQC spectrum of compounds **5** and **6**. 19
Figure S37. ^{1}H-^{1}H COSY spectrum of compounds **5** and **6**. 20
Figure S38. HMBC spectrum of compounds **5** and **6**. 20
Figure S39. ^{1}H NMR (500 MHz, DMSO-d_6) spectrum of compounds **5** and **6**. 21
Figure S40. ^{13}C NMR (125 MHz, DMSO-d_6) spectrum of compounds **5** and **6**. 21
Figure S41. HSQC spectrum of compounds **5** and **6**. 22
Figure S42. ^{1}H-^{1}H COSY spectrum of compounds **5** and **6**. 22
Figure S43. HMBC spectrum of compounds **5** and **6**. 23
Figure S44. ROESY spectrum of compounds **5** and **6**. 23
Figure S45. ESI-MS spectrum of compounds **5** and **6**. 24
Figure S46. HRESI-MS of compounds **5** and **6**. 24
Figure S47. Cell viability of compound **1** in BMDMs 26
Figure S48. Compound **5**+**6** inhibits osteoclast formation in BMDMs. 26

Table 1. ^{1}H NMR (500 MHz) data of **1**–**6** in CD_3OD.

Position	δ_H (*Int.*, *Multi.*, *J* in Hz)					
	**1**	**2**	**3**	**4**	**5**	**6**
6	6.31 (1H, s)	6.32 (1H, s)	6.33 (1H, s)	6.33 (1H, s)	6.31 (1H, s)	6.31 (1H, s)
2'	7.72 (1H, d, 2.1)	7.78 (1H, d, 2.0)	7.94 (1H, d, 9.0)	7.93 (1H, d, 9.0)	7.78 (1H, d, 2.0)	7.85 (1H, d, 2.0)
3'			6.84 (1H, d, 9.0)	6.85 (1H, d, 9.0)		
5'	6.84 (1H, d, 9.0)	6.84 (1H, d, 8.5)	6.84 (1H, d, 9.0)	6.85 (1H, d, 9.0)	6.85 (1H, d, 8.5)	6.84 (1H, d, 8.5)
6'	7.46 (1H, dd, 9.0, 2.1)	7.42 (1H, dd, 8.5, 2.0)	7.94 (1H, d, 9.0)	7.93 (1H, d, 9.0)	7.53 (1H, dd, 8.5, 2.0)	7.48 (1H, dd, 8.5, 2.0)
-OCH_3					3.82 (3H, s)	3.84 (3H, s)
1''	4.05 (2H, br. s)	4.04 (2H, s)	4.04 (2H, m)	4.05 (2H, m)	4.05 (2H, m)	4.05 (2H, m)
3'', 7''	7.07 (2H, d, 8.4)	7.07 (2H, d, 8.5)	7.04 (2H, d, 8.5)	7.05 (2H, d, 8.5)	7.04 (2H, d, 8.0)	7.03 (2H, d, 8.0)
4'', 6''	6.64 (2H, d, 8.4)	6.63 (2H, d, 8.5)	6.66 (2H, d, 8.5)	6.66 (2H, d, 8.5)	6.67 (2H, d, 8.0)	6.65 (2H, d, 8.0)
1'''	5.08 (1H, d, 7.8)	5.25 (1H, d, 7.5)	5.02 (1H, d, 7.5)	5.12 (1H, d, 7.5)	5.22 (1H, d, 7.5)	5.20 (1H, d, 7.5)
2'''	3.46 (1H, dd, 7.8, 7.8)	3.48 (1H, dd, 8.5, 7.5)	3.79 (1H, dd, 8.0, 7.5)	3.44 (1H, dd, 7.5, 7.5)	3.49 (1H, dd, 7.5, 7.5)	3.83 (1H, dd, 8.0, 7.5)
3'''	3.41 (1H, dd, 7.8, 7.8)	3.43 (1H, dd, 8.5, 8.5)	3.55 (1H, dd, 8.0, 2.5)	3.41 (1H, dd, 7.5, 8.0)	3.46 (1H, dd, 7.5, 8.0)	3.54 (1H, dd, 8.0, 2.5)
4'''	3.29 (1H, dd, 7.8, 7.8)	3.36 (1H, dd, 8.5, 8.5)	3.76 (1H, dd, 3.5, 2.5)	3.28 (1H, dd, 8.0, 8.0)	3.29 (1H, dd, 8.0, 8.0)	3.75 (1H, dd, 3.5, 2.5)
5'''	3.30 (1H, m)	3.22 (1H, m)	3.62 (1H, m)	3.34 (1H, m)	3.38 (1H, m)	3.66 (1H, m)
6'''a	3.79 (1H, dd, 12.4, 1.2)	3.71 (1H, dd, 12.0, 2.5)	3.74 (1H, dd, 11.5, 6.0)	3.80 (1H, dd, 11.0, 1.5)	3.81 (1H, dd, 11.5, 1.5)	3.76 (1H, dd, 11.5, 6.0)
6'''b	3.38 (1H, dd, 12.4, 5.4)	3.58 (1H, dd, 12.0, 5.0)	3.36 (1H, dd, 11.5, 6.5)	3.39 (1H, dd, 11.0, 6.0)	3.42 (1H, dd, 11.0, 6.0)	3.36 (1H, dd, 11.5, 6.5)
1''''	4.52 (1H, d, 1.2)		4.52 (1H, d, 1.5)	4.52 (1H, d, 1.5)	4.54 (1H, d, 1.5)	4.53 (1H, d, 1.5)
2''''	3.64 (1H, dd, 3.0, 1.2)		3.61 (1H, dd, 3.0, 1.5)	3.65 (1H, dd, 3.5, 1.5)	3.64 (1H, dd, 3.5, 1.5)	3.61 (1H, dd, 3.0, 1.5)
3''''	3.56 (1H, dd, 9.0, 3.0)		3.52 (1H, dd, 9.0, 3.0)	3.55 (1H, dd, 9.5, 3.5)	3.52(1H, dd, 9.5, 3.5)	3.52 (1H, dd, 9.0, 3.0)
4''''	3.28 (1H, dd, 9.0, 9.0)		3.30 (1H, dd, 9.0, 9.0)	3.30 (1H, dd, 9.5, 9.5)	3.27 (1H, dd, 9.5, 9.5)	3.30 (1H, dd, 9.0, 9.0)
5''''	3.44 (1H, m)		3.53 (1H, m)	3.47 (1H, m)	3.40 (1H, m)	3.53 (1H, m)
6''''	1.12 (3H, d, 6.6)		1.20 (1H, d, 6.5)	1.13 (1H, d, 6.0)	1.11 (1H, d, 6.0)	1.19 (1H, d, 6.5)

Table S2. ^{13}C NMR (125 MHz) data of 1–6 in CD_3OD.

Position	δC					
	1	2	3	4	5	6
2	159.5	157.5	157.9	157.9	157.3	157.3
3	135.8	134.1	134.3	134.1	134.1	134.1
4	179.8	178.3	178.4	178.2	178.1	178.3
5	161.0	159.5	159.5	159.5	159.5	159.5
6	99.6	98.0	98.1	98.1	98.1	98.1
7	163.9	162.3	162.3	162.3	162.4	162.4
8	108.4	106.8	106.6	106.6	106.3	106.3
9	156.0	154.4	154.5	154.5	154.5	154.5
10	105.8	104.3	104.2	104.3	104.3	104.3
1'	123.4	121.7	121.3	121.4	121.6	121.6
2'	118.2	116.5	131.1	131.0	112.7	112.8
3'	145.9	144.5	114.6 [a]	114.7	146.9	147.0
4'	150.0	148.5	160.1	160.0	149.4	149.4
5'	116.1	114.5	114.6 [a]	114.7	114.6	114.5
6'	123.6	121.8	131.1	131.0	123.0	122.9
-OCH_3					55.3	55.4
1''	28.4	26.8	26.8	26.8	26.7	26.7
2'	133.2	131.6	131.5	131.6	131.3	131.3
3'', 7''	130.4	128.8	128.7	128.7	128.6	128.7
4'', 6''	116.2	114.6	114.6[a]	114.6	114.7	114.7
5''	156.5	155.6	155.0	155.0	155.0	155.0
1'''	105.2	103.0	104.4	103.4	103.3	104.4
2'''	75.9	74.3	71.6	74.4	74.5	71.7
3'''	78.4	76.7	73.7	76.8	76.8	73.6
4'''	71.4	69.8	68.7	69.9	70.0	68.6
5'''	77.3	77.0	73.8	75.8	75.9	73.9
6'''	68.6	61.1	65.8	65.8	67.0	65.8
1''''	102.5		100.4	101.0	101.0	100.4
2''''	72.2		70.6	70.7	70.6	70.6
3''''	72.4		70.9	70.9	70.9	70.9
4''''	74.1		72.5	72.5	72.5	72.5
5''''	69.8		68.3	68.3	68.3	68.3
6''''	18.0		16.6	16.5	16.5	16.6

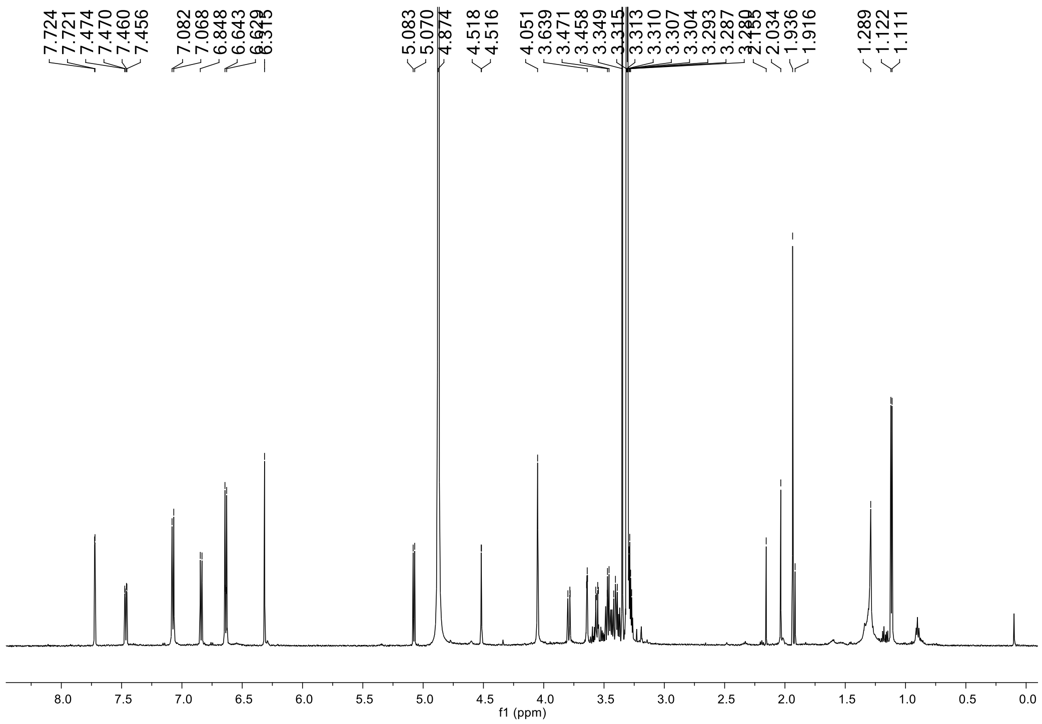

Figure S1. ^{1}H NMR (500 MHz, CD_3OD) spectrum of compound **1**.

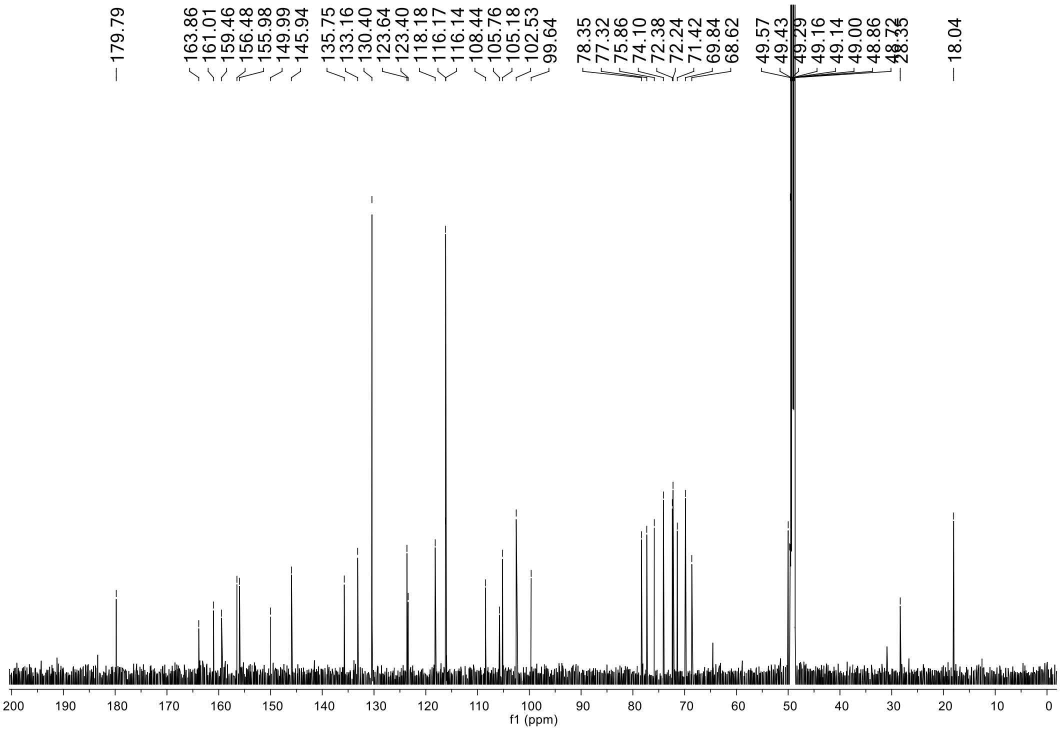

Figure S2. ^{13}C NMR (125 MHz, CD_3OD) spectrum of compound **1**.

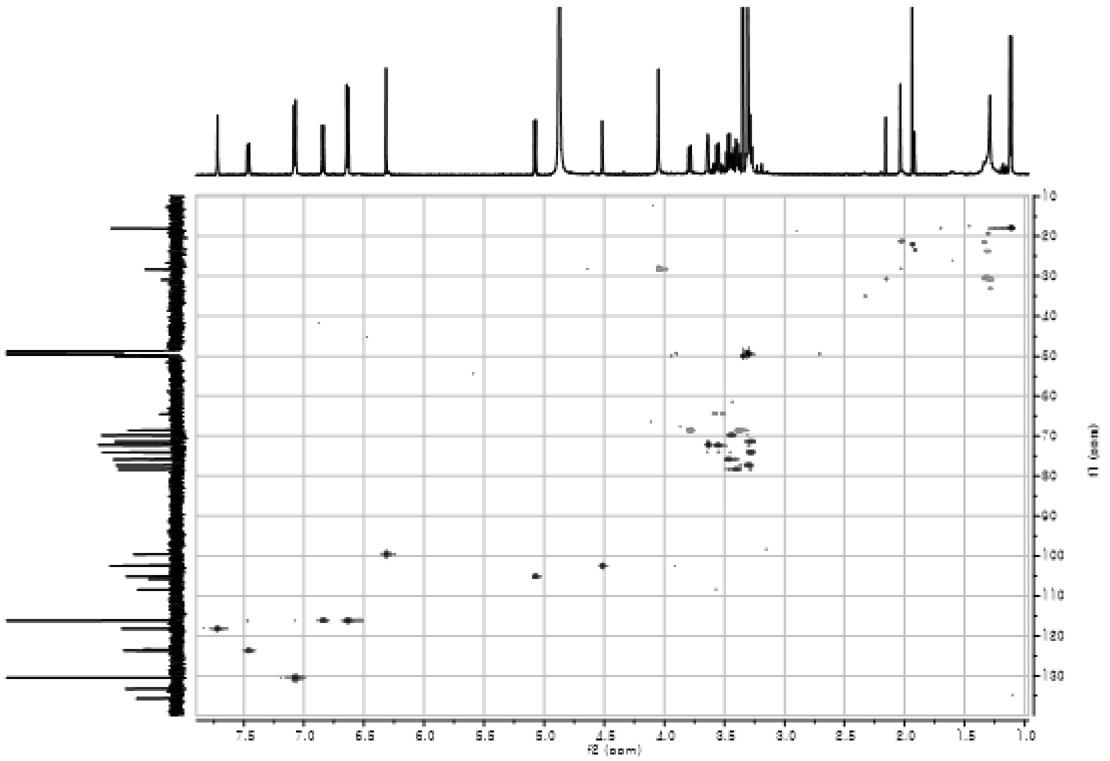

Figure S3. HSQC spectrum of compound **1**.

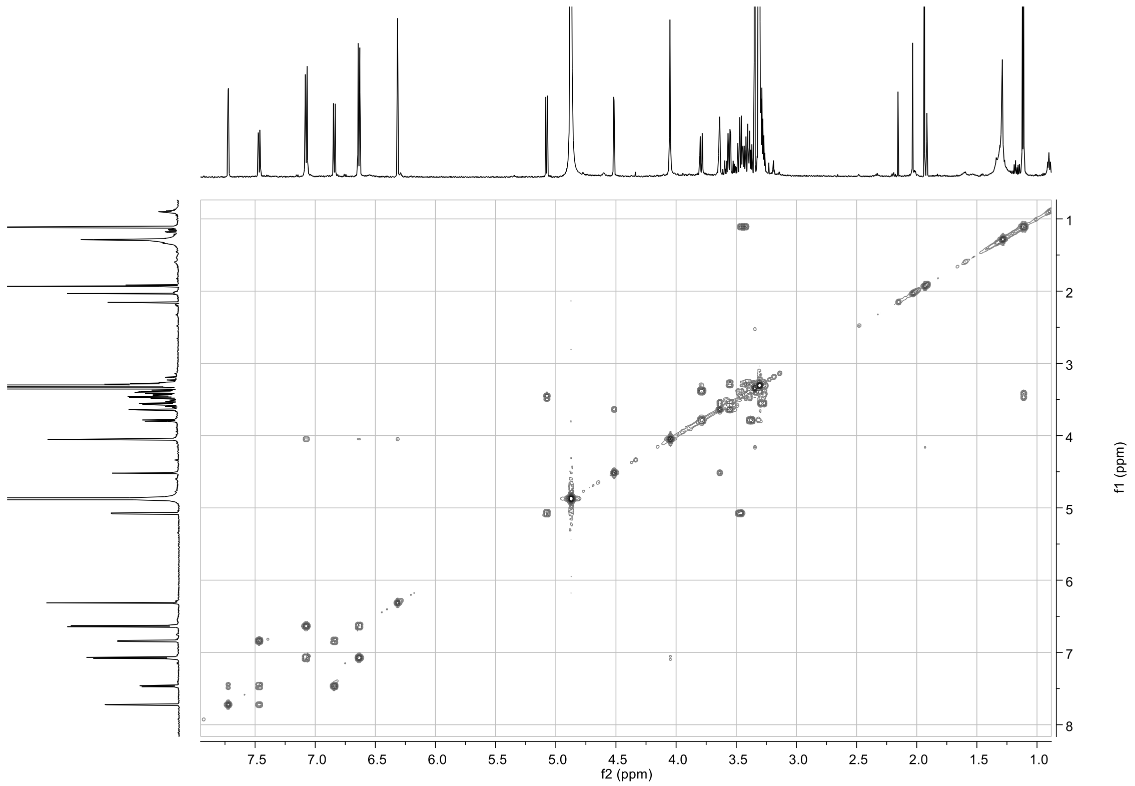

Figure S4. ^{1}H-^{1}H COSY spectrum of compound **1**.

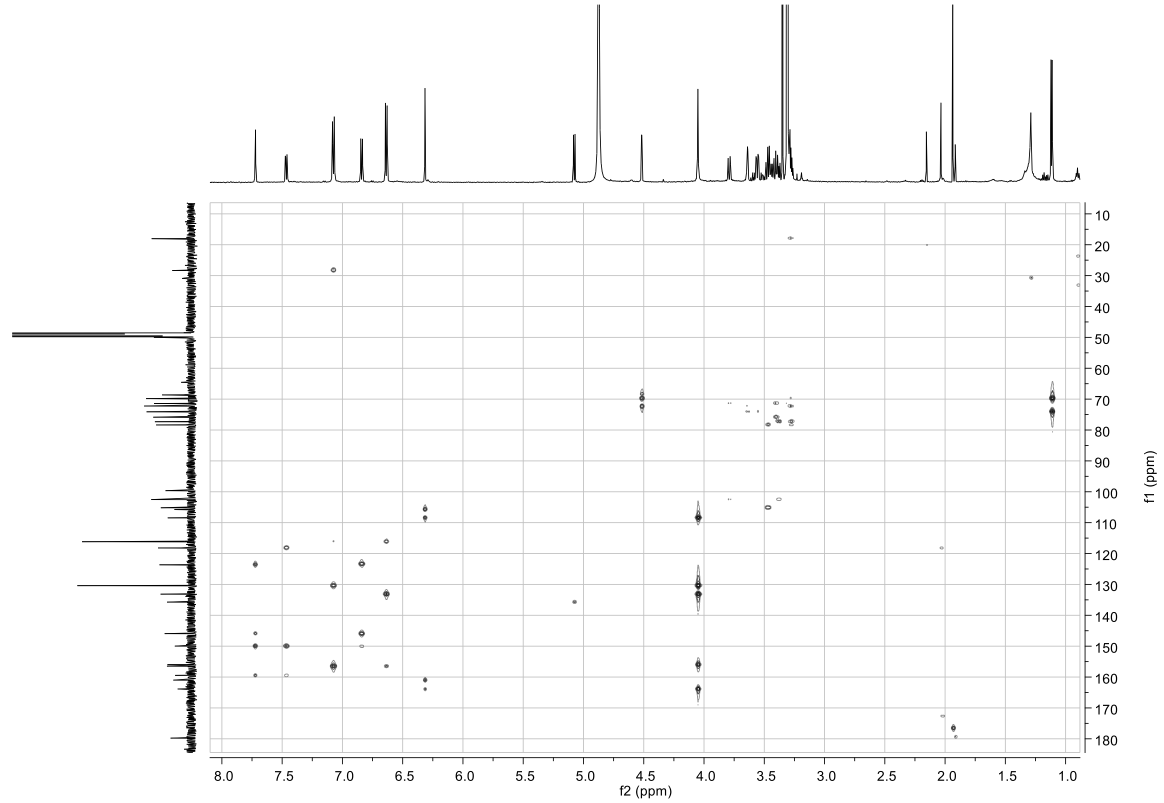

Figure S5. HMBC spectrum of compound **1**.

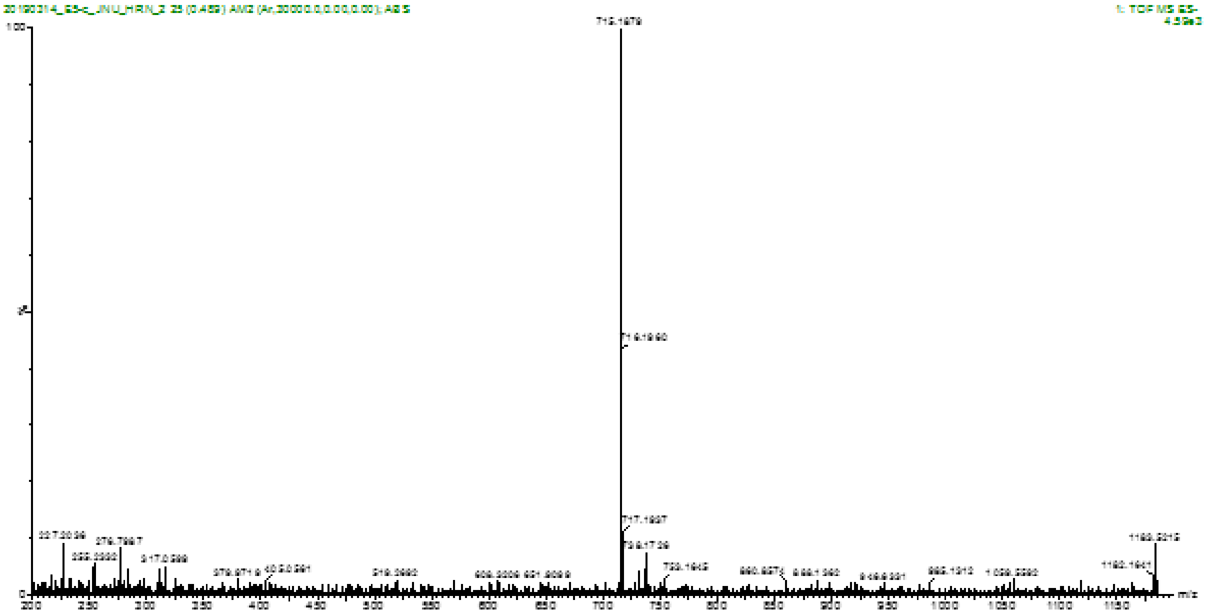

Figure S6. ESI-MS spectrum of compound **1**.

Elemental Composition Report

Single Mass Analysis

Tolerance = 10.0 PPM / DBE: min = -1.5, max = 50.0

Element prediction: Off

Number of isotope peaks used for i-FIT = 3

Monoisotopic Mass, Even Electron Ions

100 formula(e) evaluated with 3 results within limits (all results (up to 1000) for each mass)

Elements Used:

C: 1-50 H: 1-50 O: 1-30

Minimum: -1.5

Maximum: 500.0 10.0 50.0

Mass	Calc. Mass	mDa	PPM	DBE	i-FIT	Norm	Conf(%)	Formula
715.1879	715.1874	0.5	0.7	17.5	205.1	1.717	17.97	C34 H35 O17

Figure S7. HRESI-MS data of compound **1**.

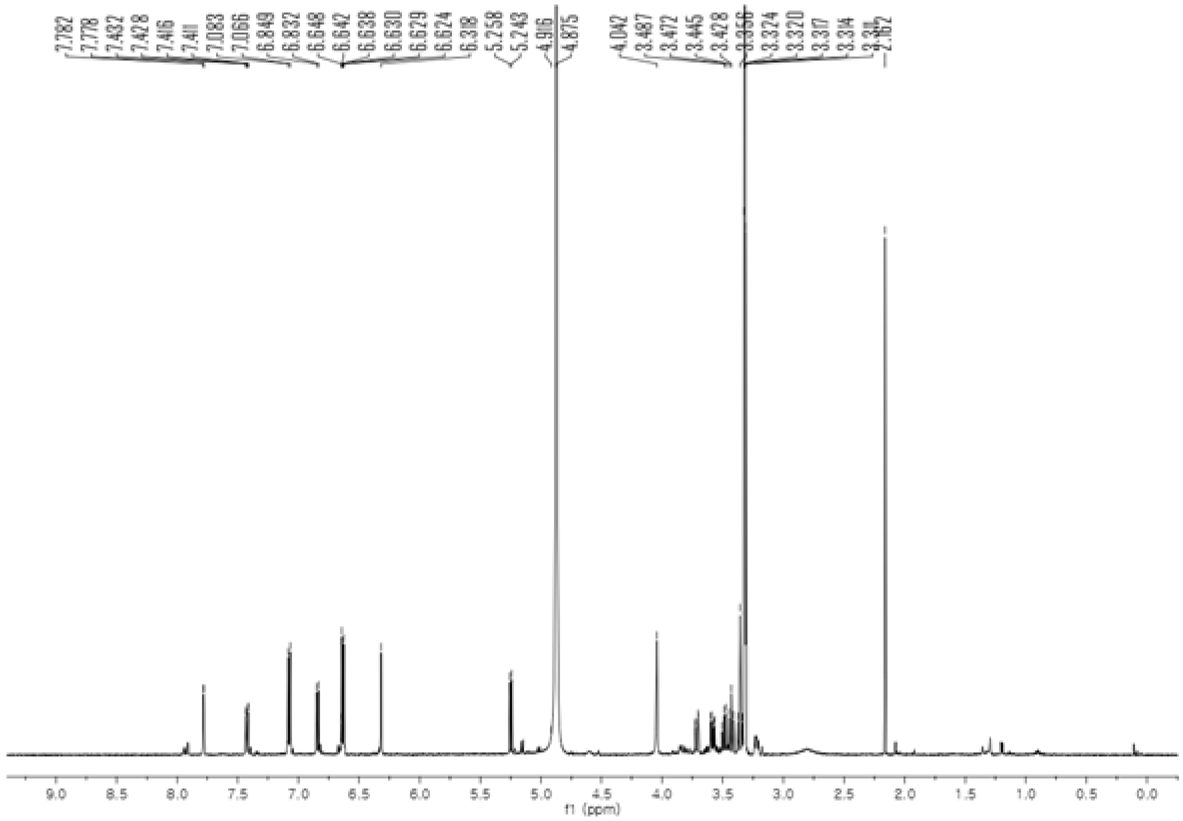

Figure S8. ^{1}H NMR (500 MHz, CD_3OD) spectrum of compound **2**.

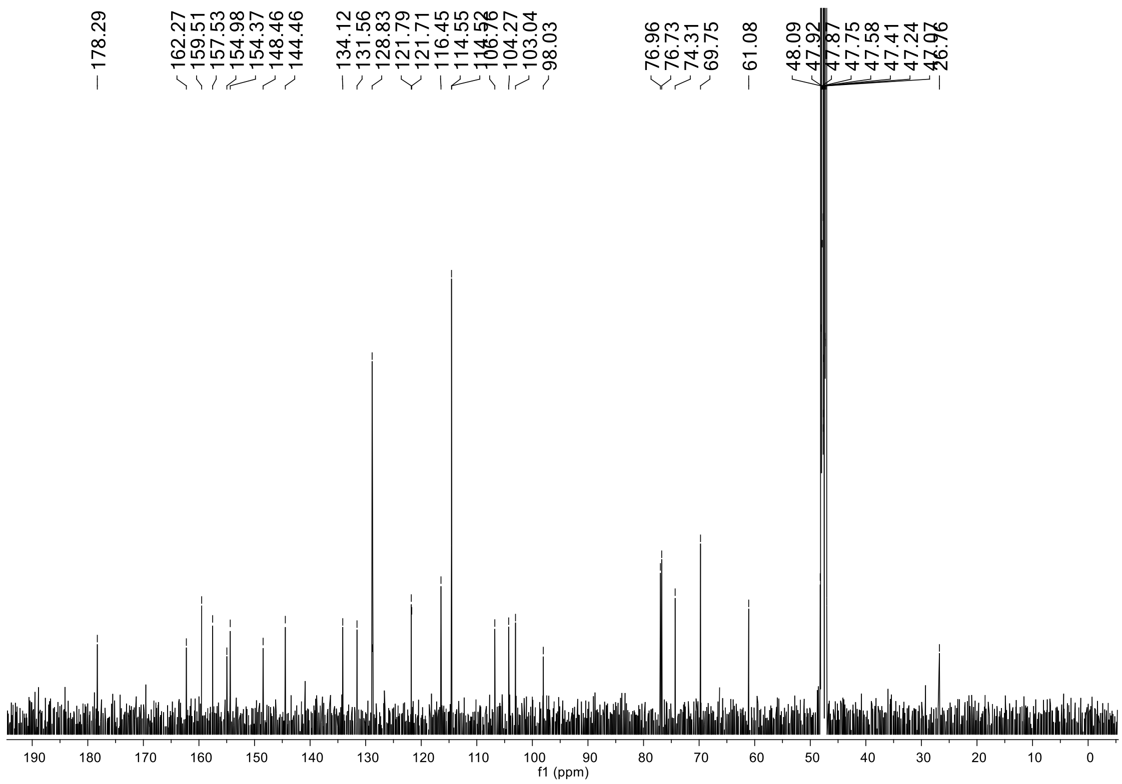

Figure S9. ^{13}C NMR (125 MHz, CD_3OD) spectrum of compound **2**.

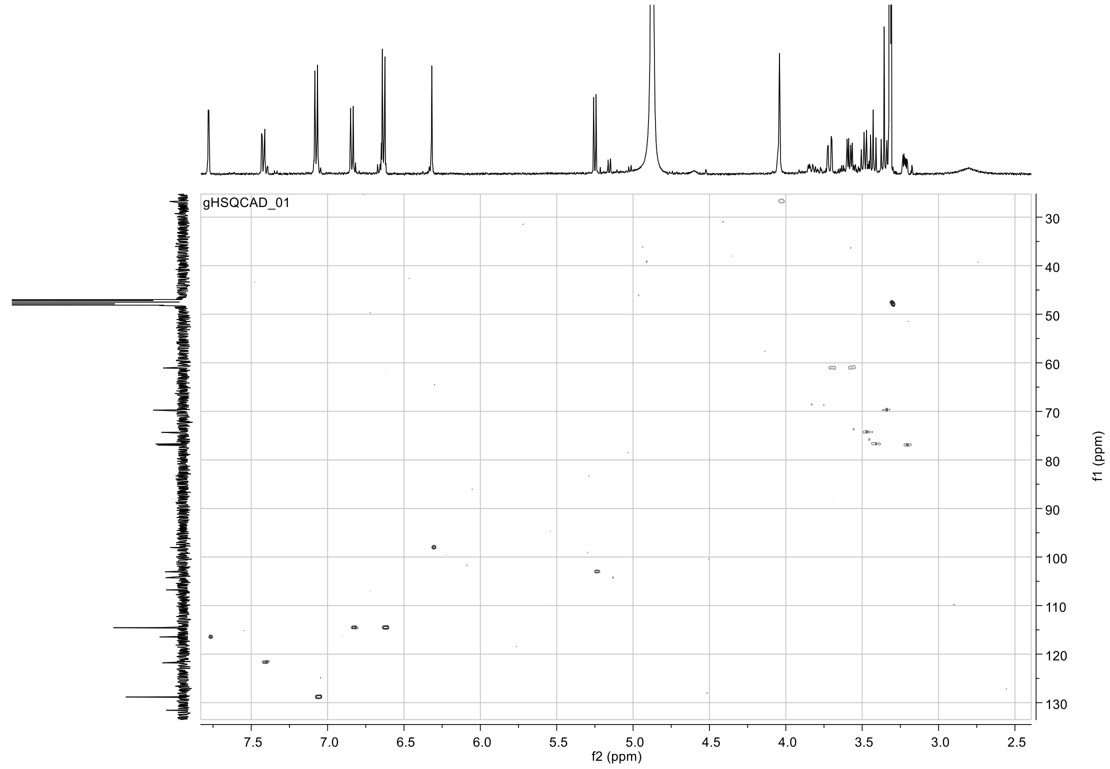

Figure S10. HSQC spectrum of compound **2**.

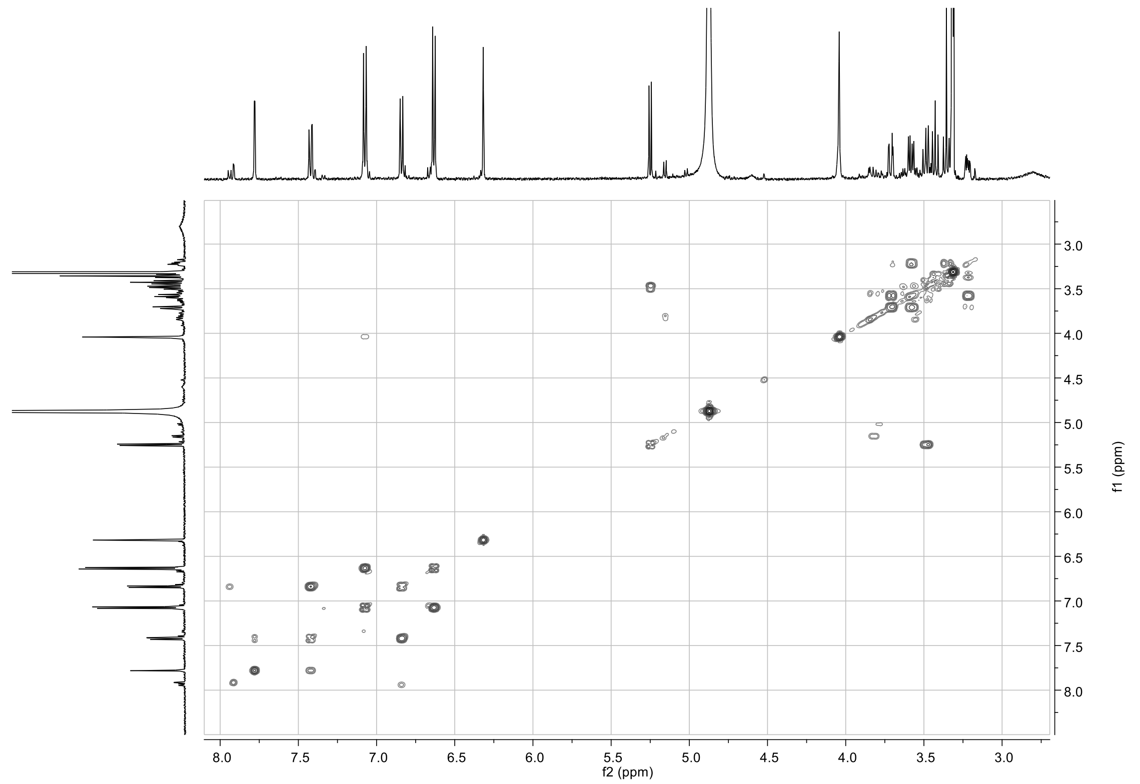

Figure S11. ^{1}H-^{1}H COSY spectrum of compound **2**.

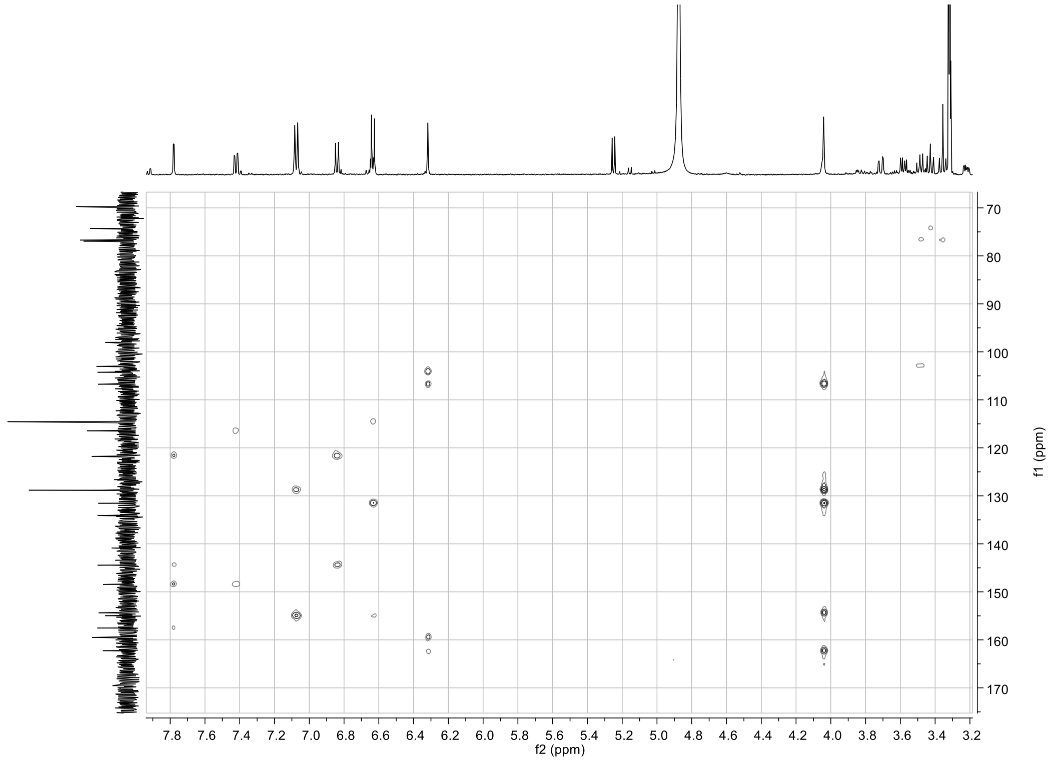

Figure S12. HMBC spectrum of compound **2**.

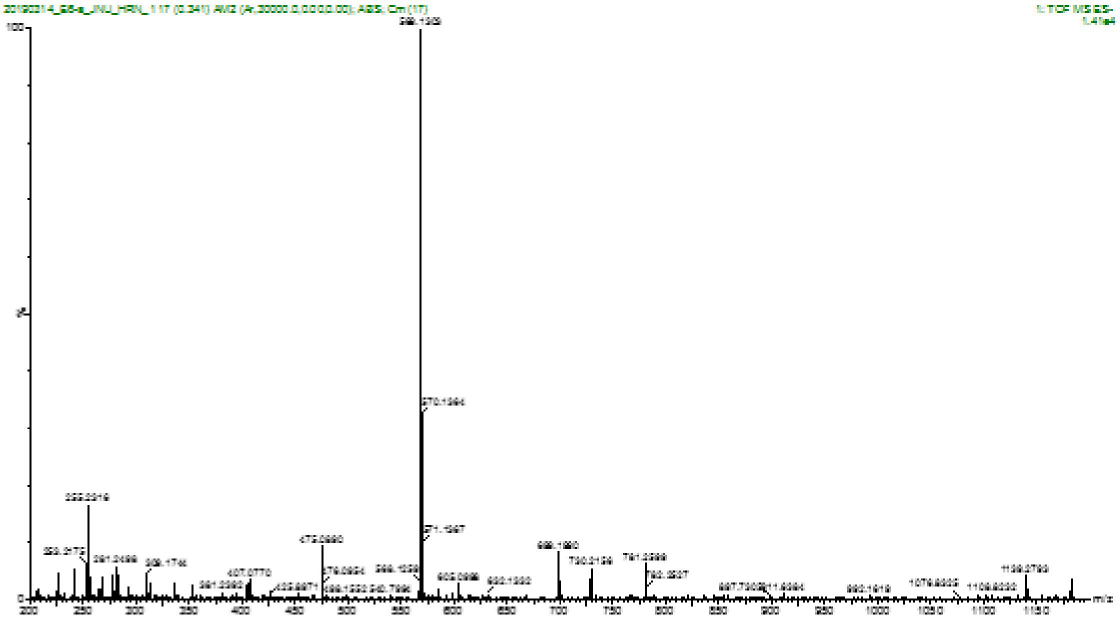

Figure S13. ESI-MS spectrum of compound **2**.

Elemental Composition Report

Single Mass Analysis

Tolerance = 10.0 PPM / DBE: min = -1.5, max = 50.0

Element prediction: Off

Number of isotope peaks used for i-FIT = 3

Monoisotopic Mass, Even Electron Ions

115 formula(e) evaluated with 2 results within limits (all results (up to 1000) for each mass)

Elements Used:

C: 1-50 H: 1-50 O: 1-30

Minimum:					-1.5			
Maximum:			500.0	10.0	50.0			
Mass	Calc. Mass	mDa	PPM	DBE	i-FIT	Norm	Conf(%)	Formula
569.1303	569.1295	0.8	1.4	16.5	362.7	0.045	95.64	C28 H25 O13

Figure S14. HRESI-MS of compound **2**.

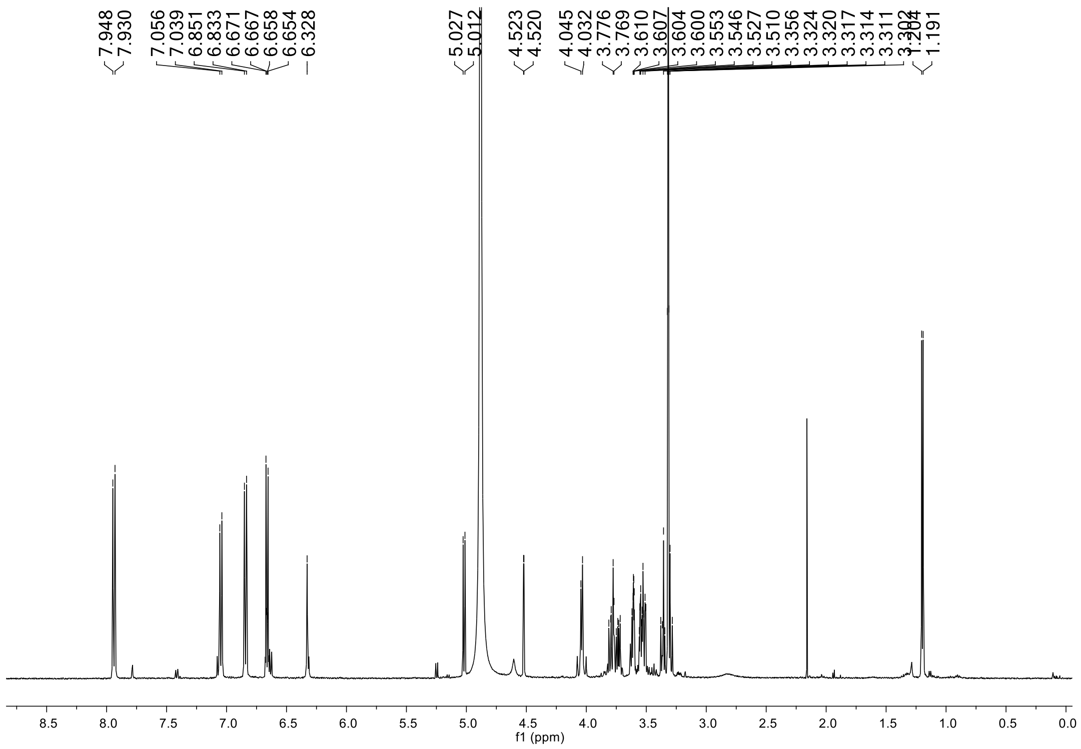

Figure S15. ^{1}H NMR (500 MHz, CD_3OD) spectrum of compound **3**.

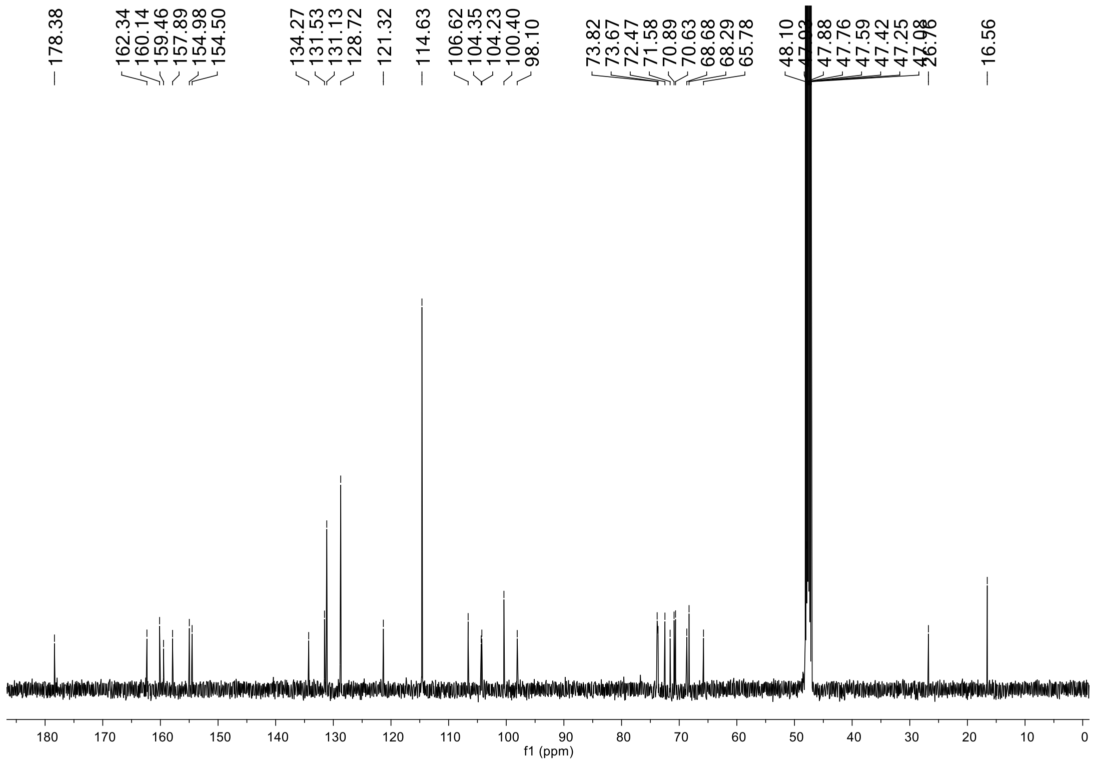

Figure S16. ^{13}C NMR (125 MHz, CD_3OD) spectrum of compound **3**.

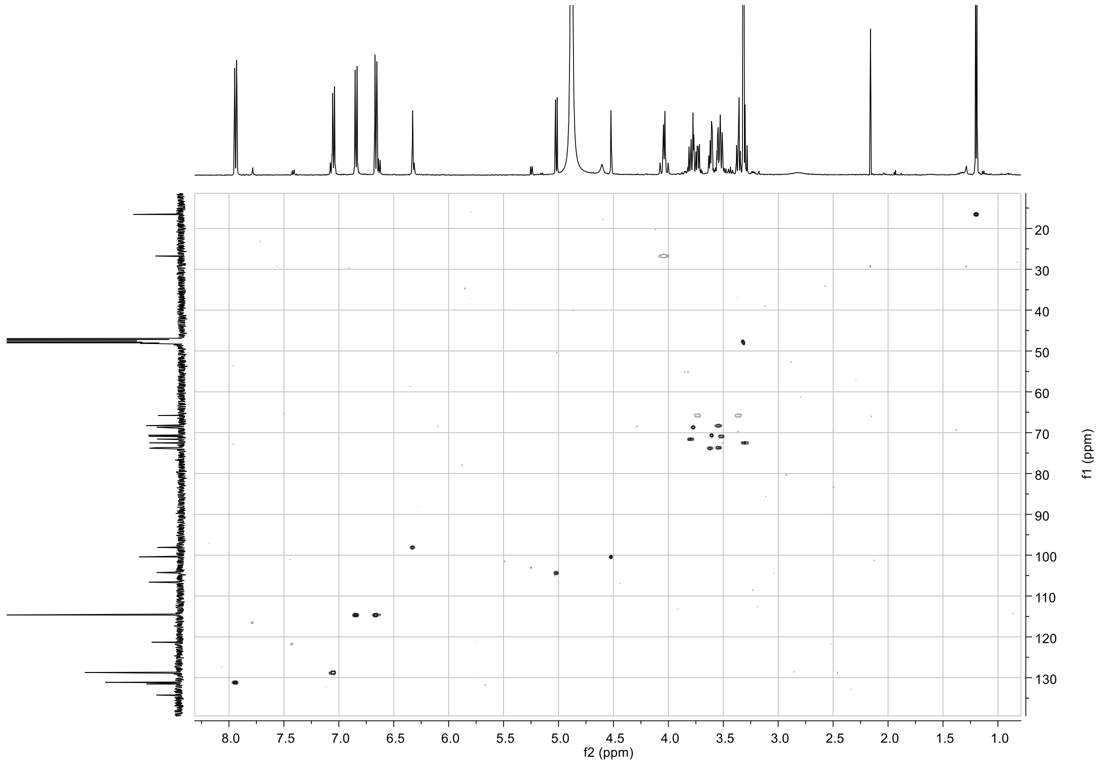

Figure S17. HSQC spectrum of compound **3**.

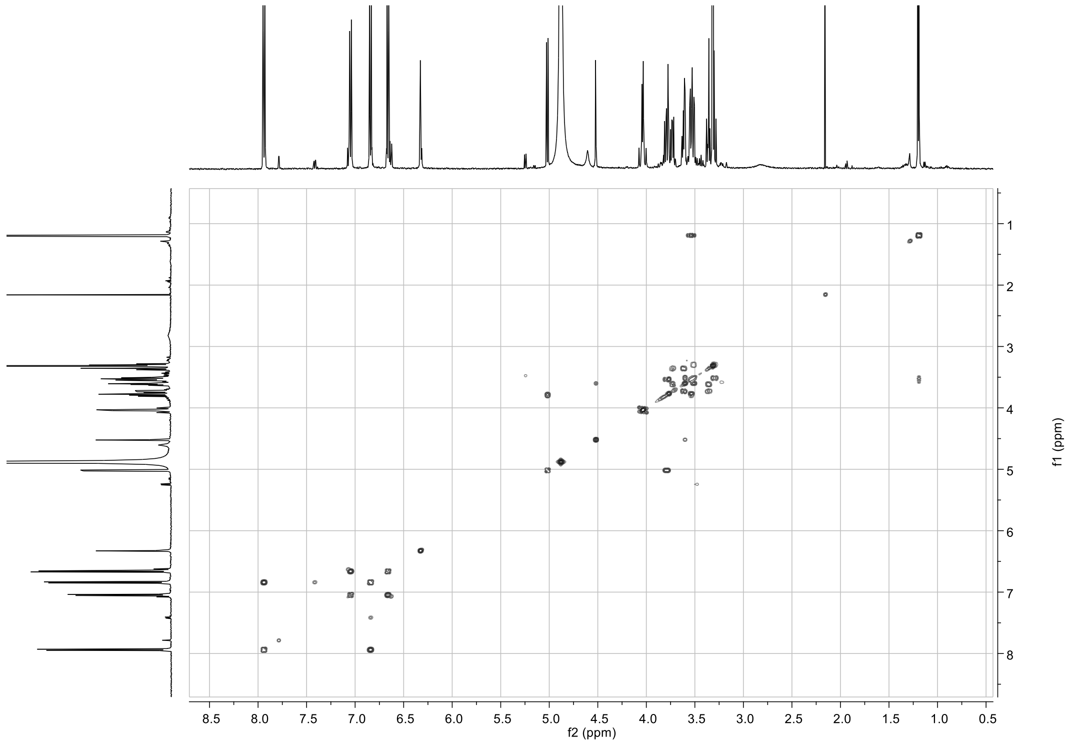

Figure S18. ^{1}H-^{1}H COSY spectrum of compound **3**.

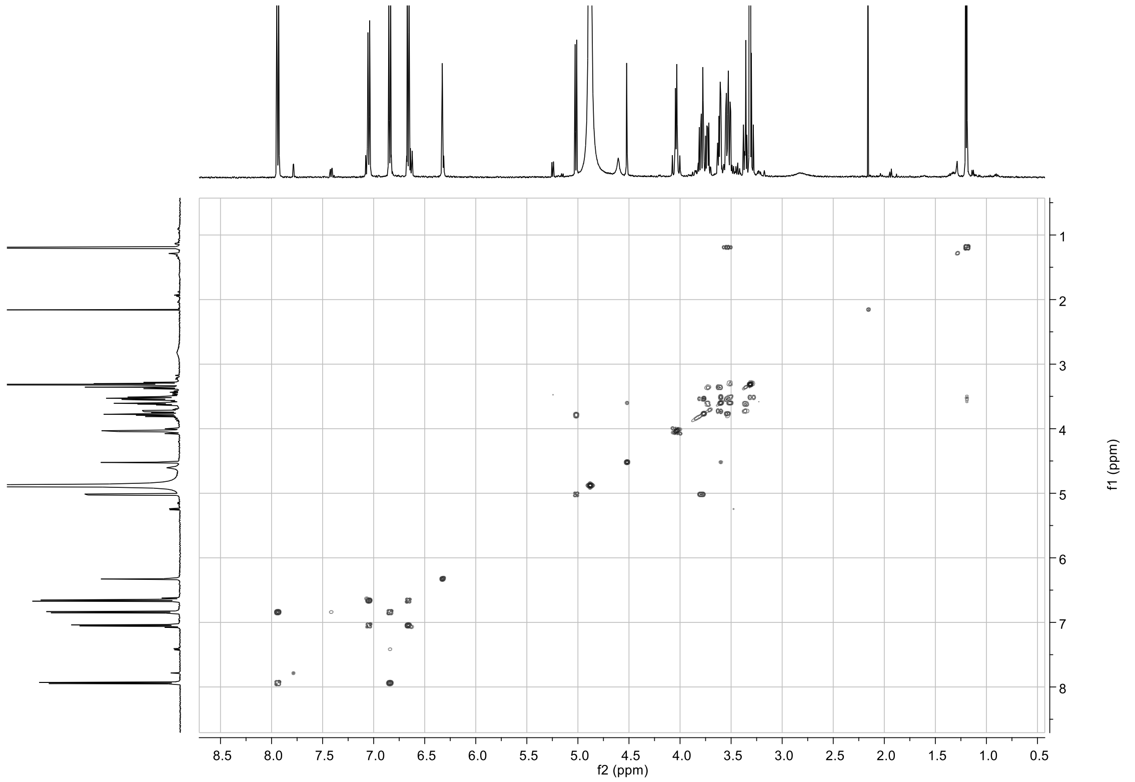

Figure S19. HMBC spectrum of compound **3**.

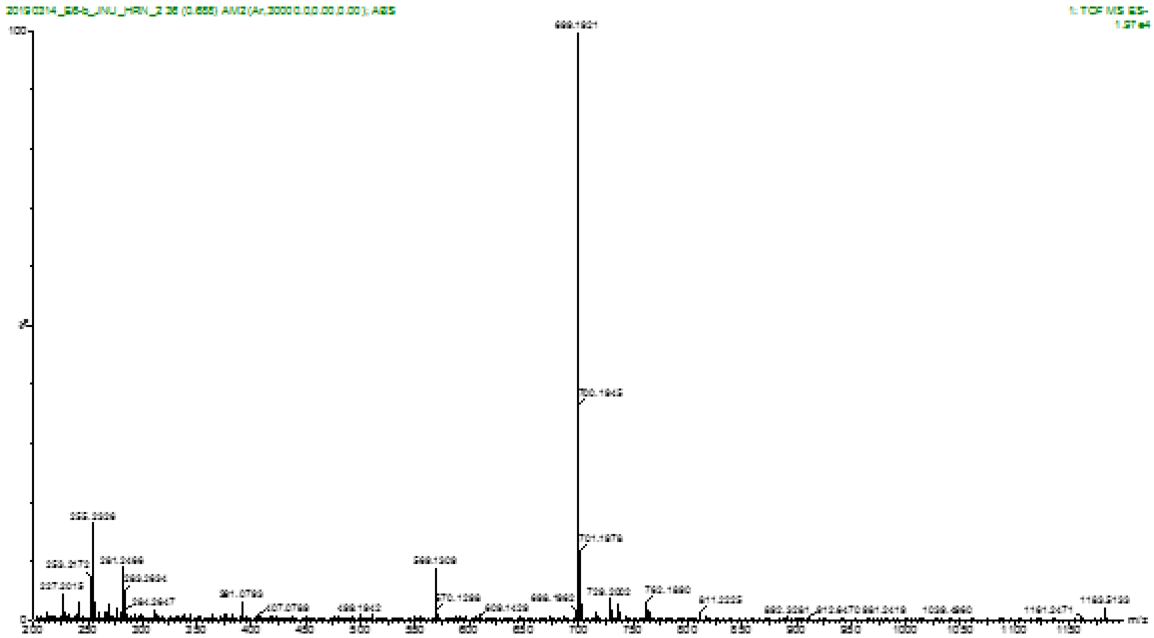

Figure S20. ESI-MS spectrum of compound **3**.

Elemental Composition Report

Single Mass Analysis

Tolerance = 10.0 PPM / DBE: min = -1.5, max = 50.0

Element prediction: Off

Number of isotope peaks used for i-FIT = 3

Monoisotopic Mass, Even Electron Ions

100 formula(e) evaluated with 3 results within limits (all results (up to 1000) for each mass)

Elements Used:

C: 1-50 H: 1-50 O: 1-30

Minimum:					-1.5			
Maximum:			500.0	10.0	50.0			
Mass	Calc. Mass	mDa	PPM	DBE	i-FIT	Norm	Conf(%)	Formula
699.1921	699.1925	-0.4	-0.6	17.5	308.2	0.009	99.11	C34 H35 O16

Figure S21. HRESI-MS data of compound **3**.

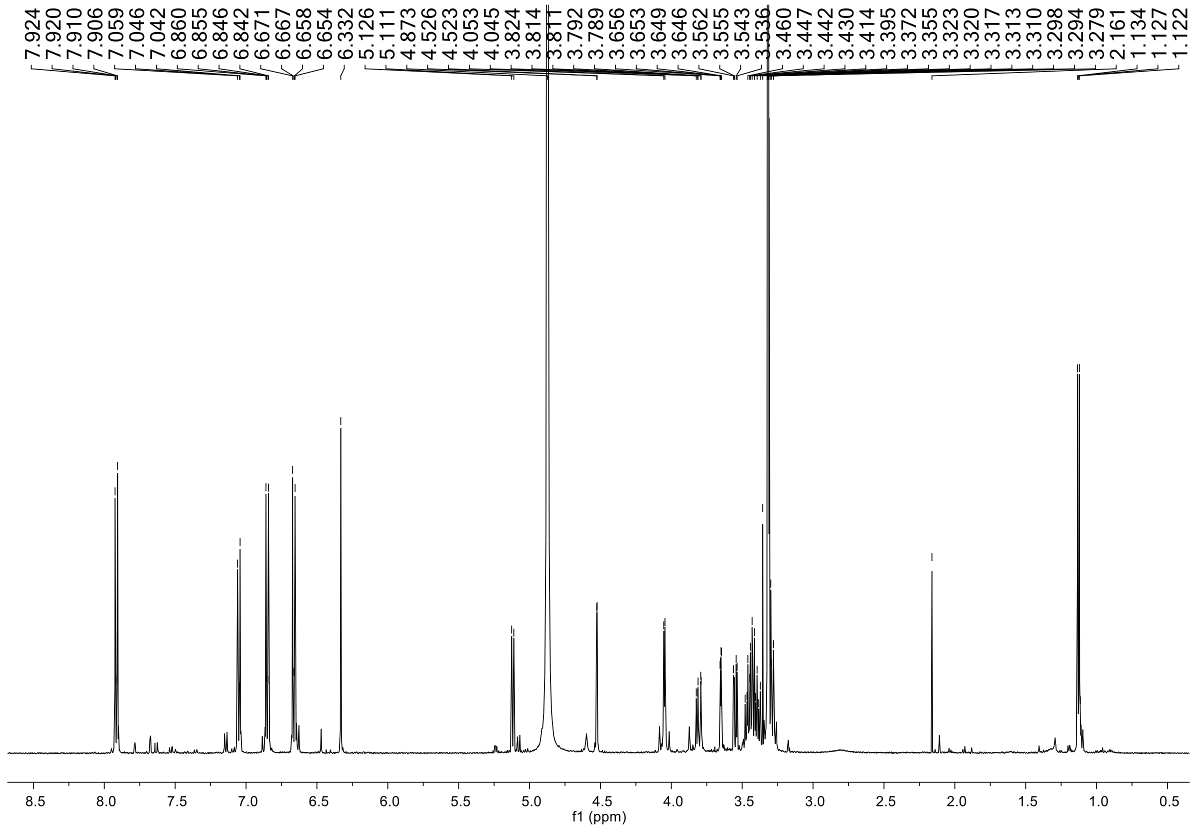

Figure S22. ^{1}H NMR (500 MHz, CD_3OD) spectrum of compound **4**.

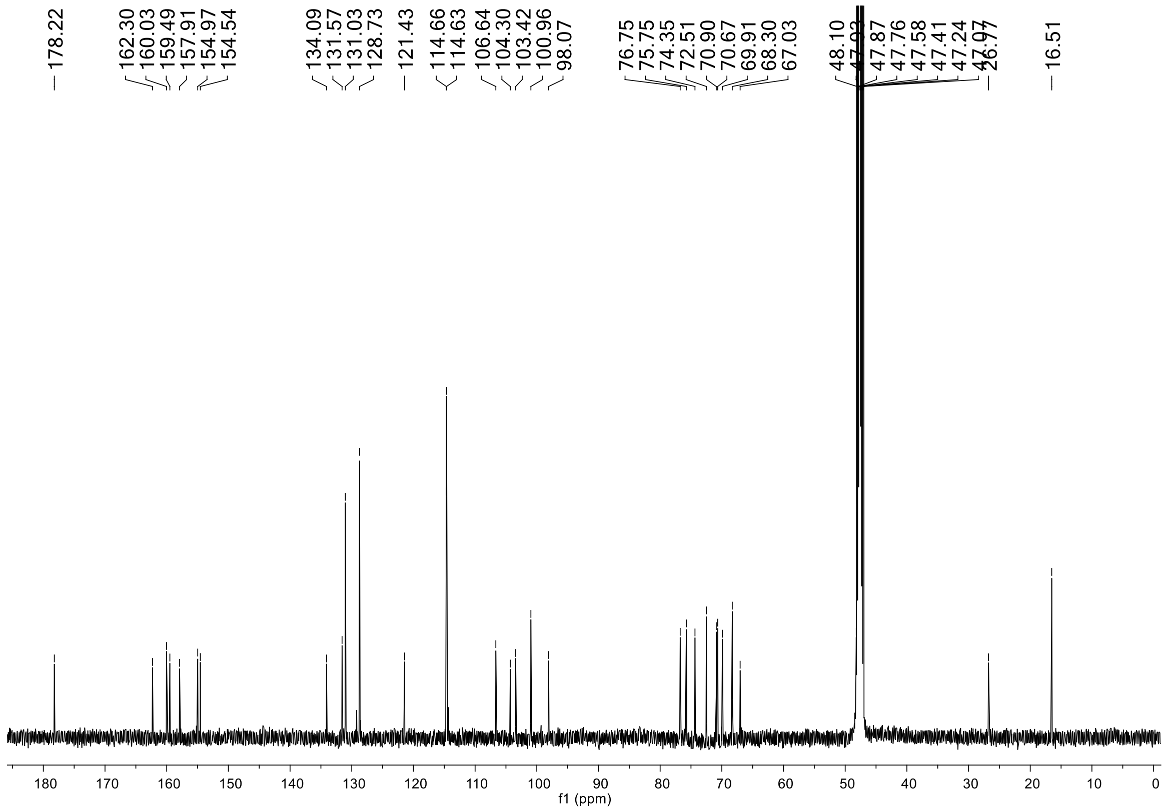

Figure S23. ^{13}C NMR (125 MHz, CD_3OD) spectrum of compound **4**.

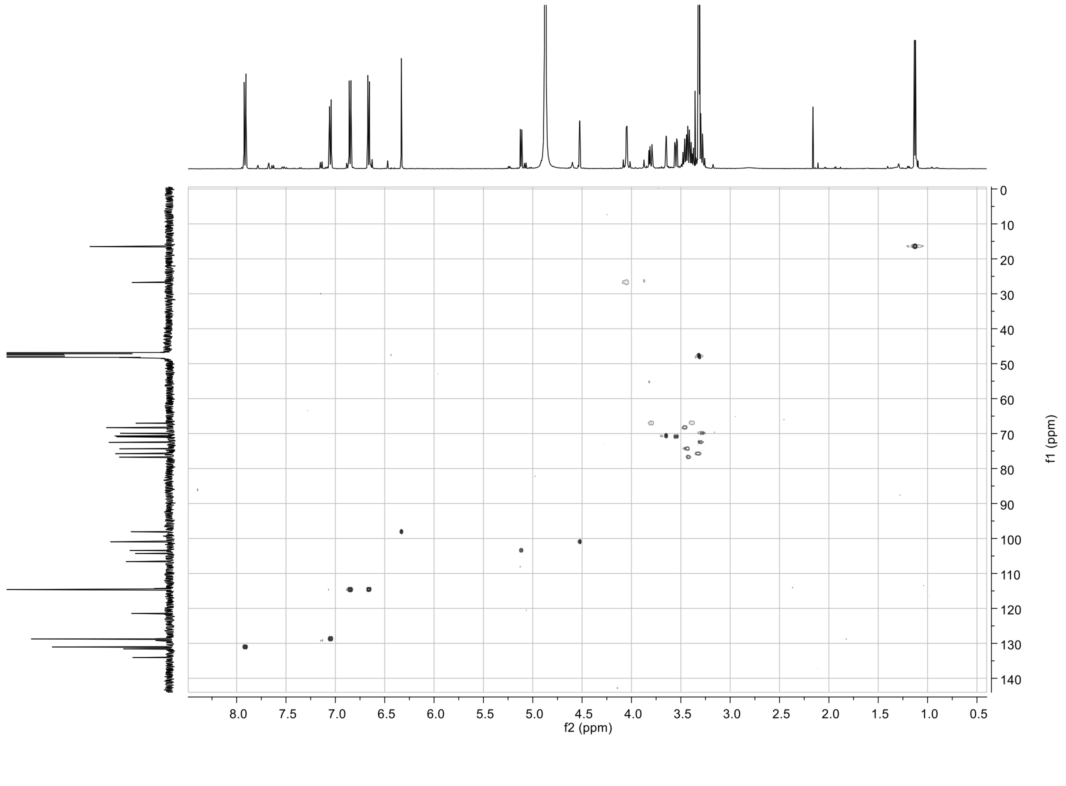

Figure S24. HSQC spectrum of compound **4**.

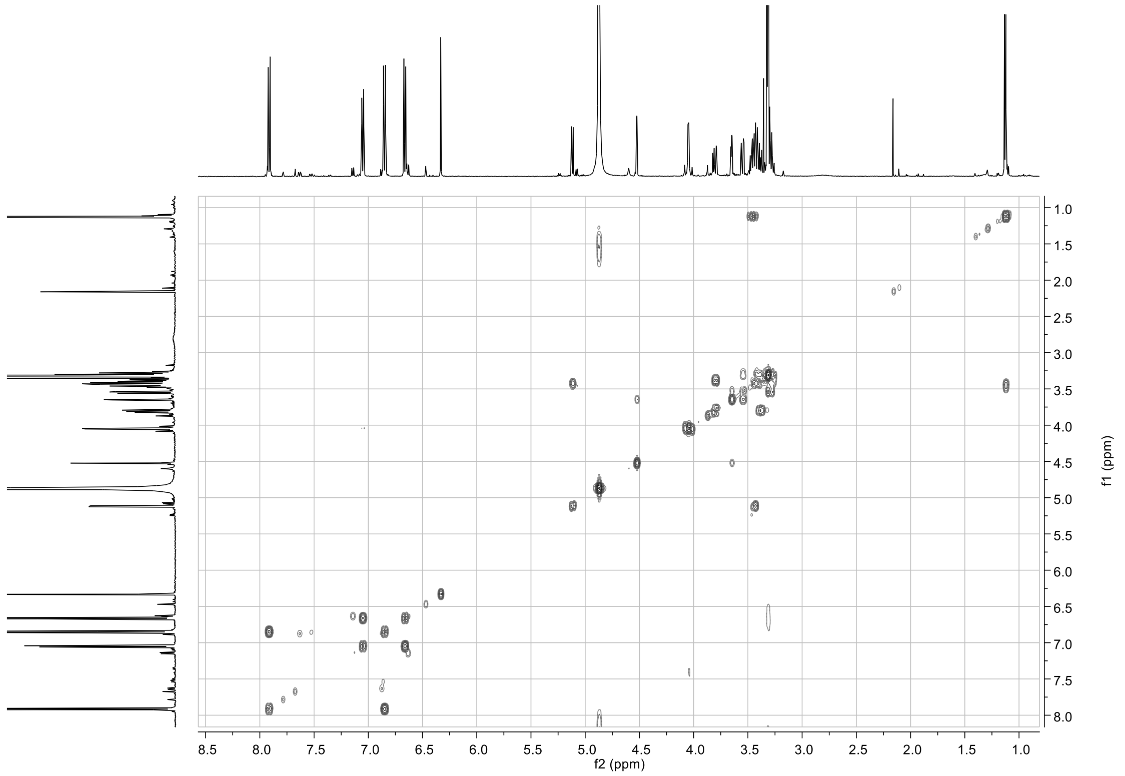

Figure S25. ^{1}H-^{1}H COSY spectrum of compound **4**.

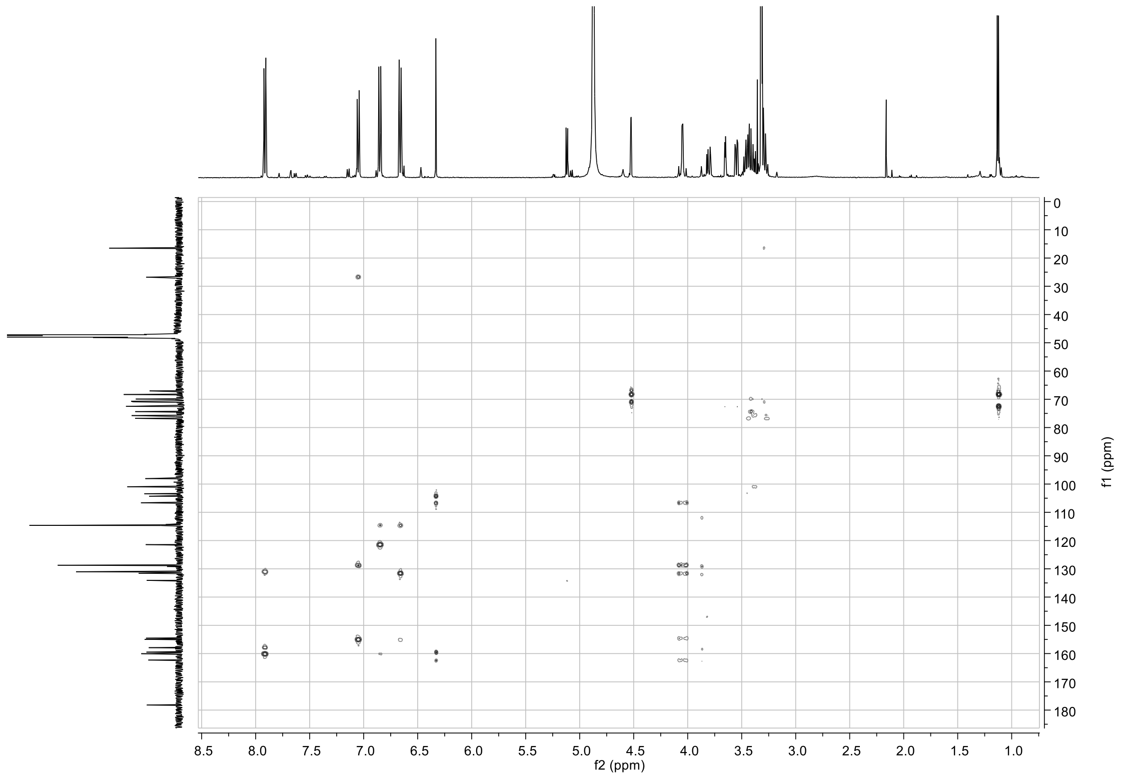

Figure S26. HMBC spectrum of compound **4**.

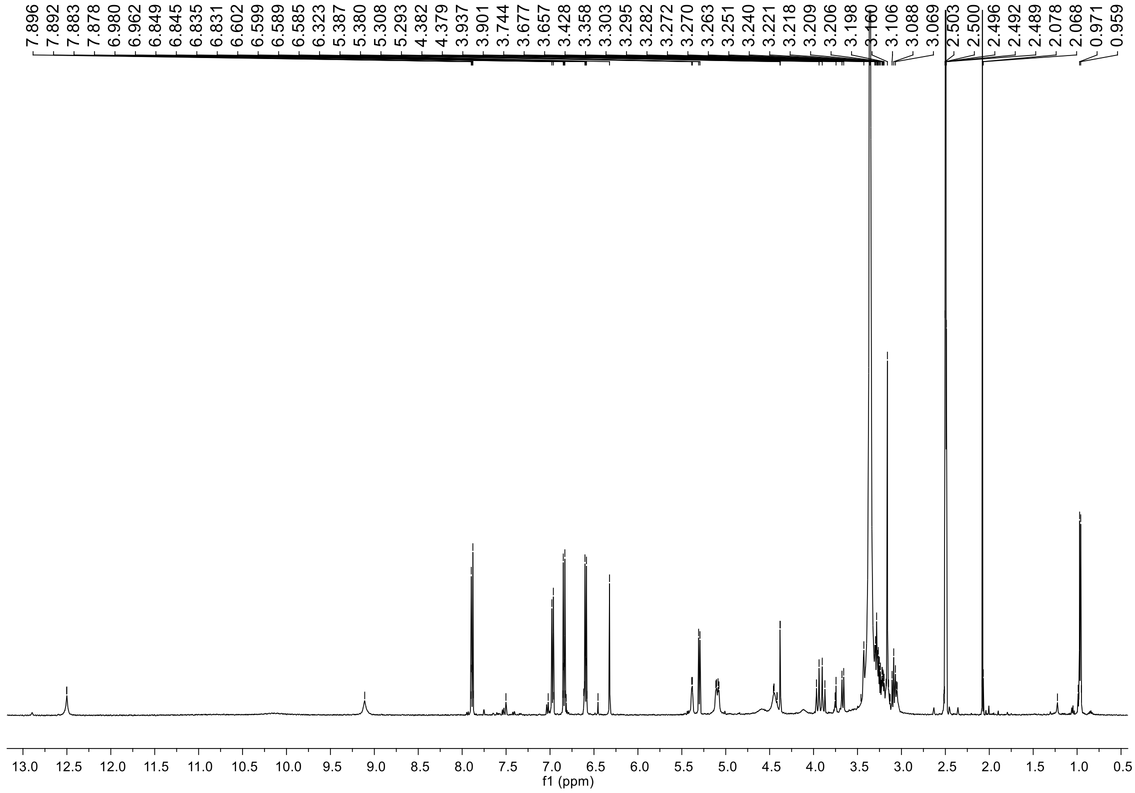

Figure S27. ^{1}H NMR (500 MHz, DMSO-d_6) spectrum of compound **4**.

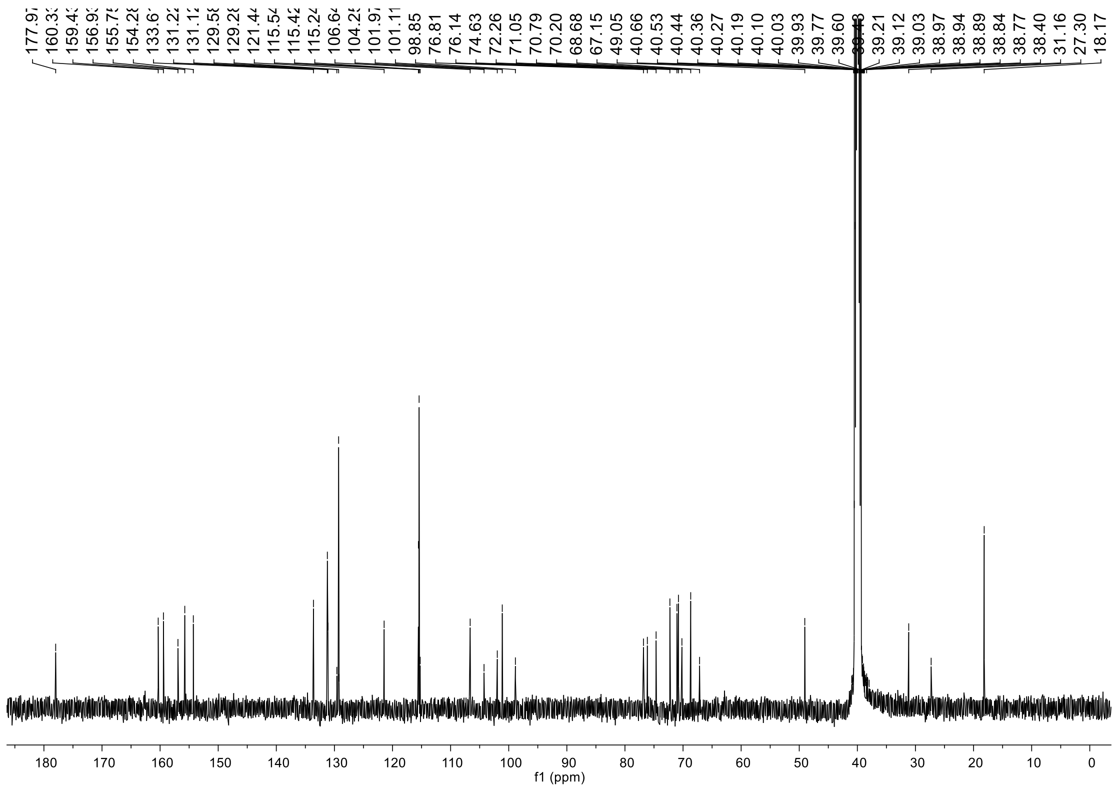

Figure S28. ^{13}C NMR (125 MHz, DMSO-d_6) spectrum of compound **4**.

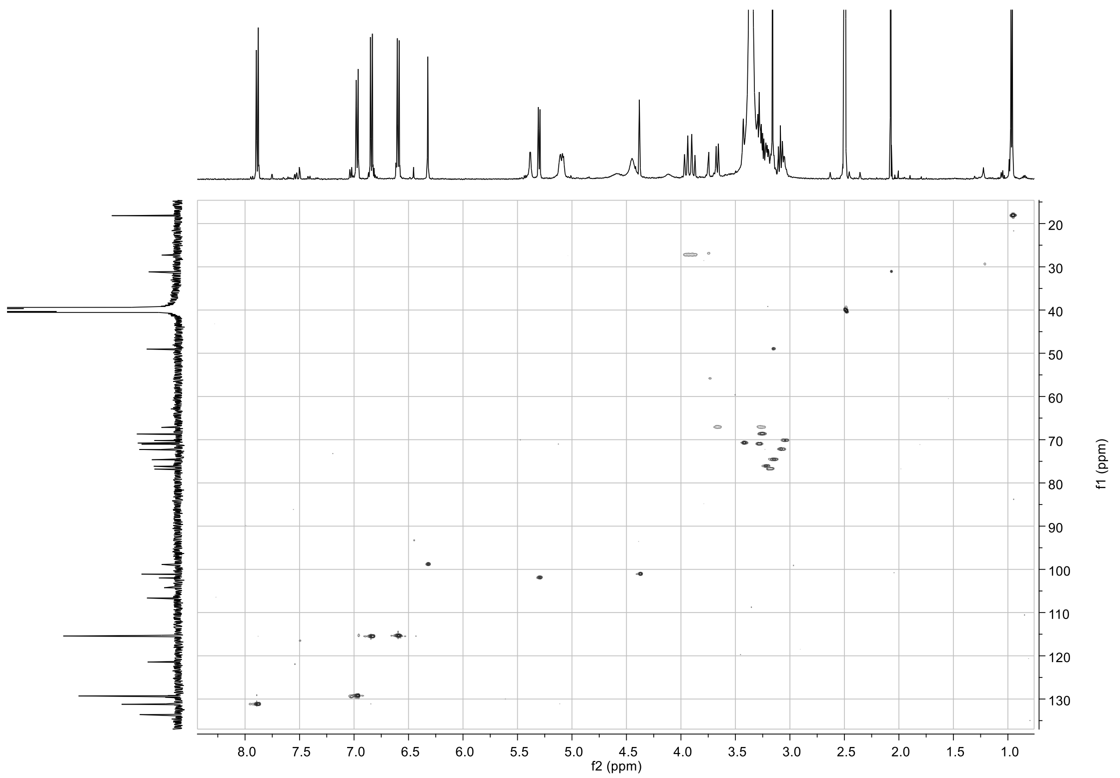

Figure S29. HSQC spectrum of compound **4**.

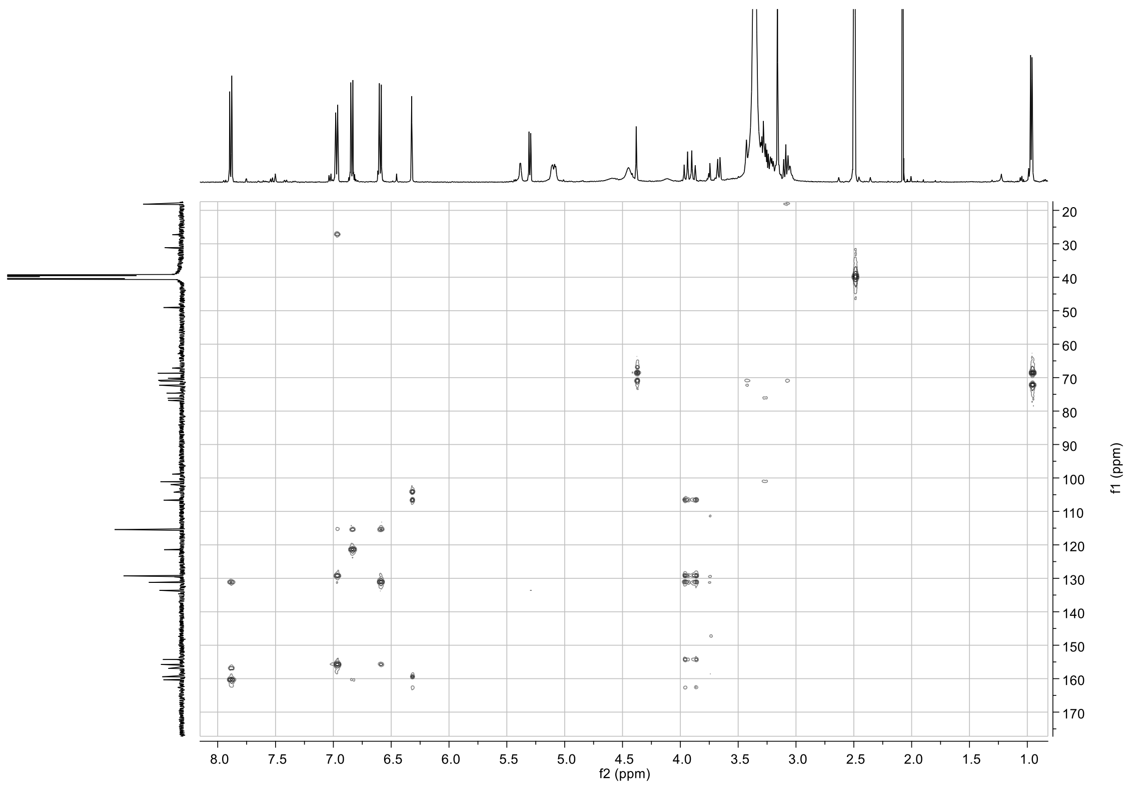

Figure S30. HMBC spectrum of compound **4**.

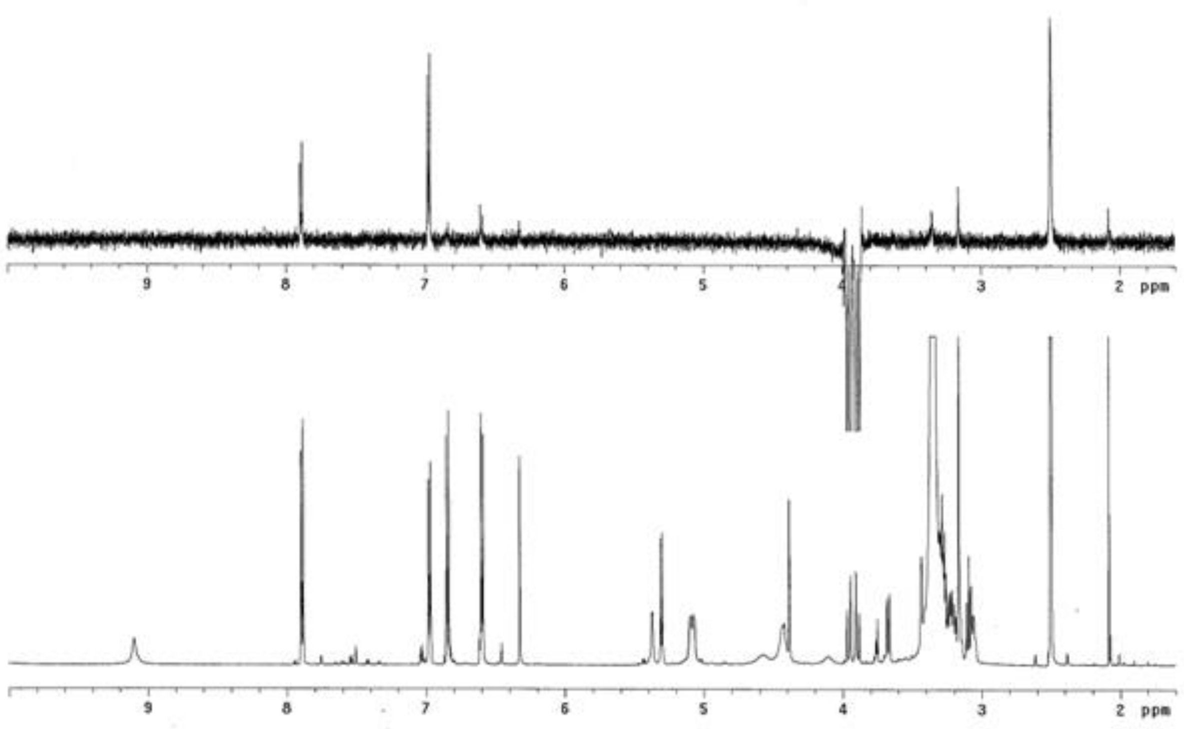

Figure S31. ROESY spectrum of compound **4**.

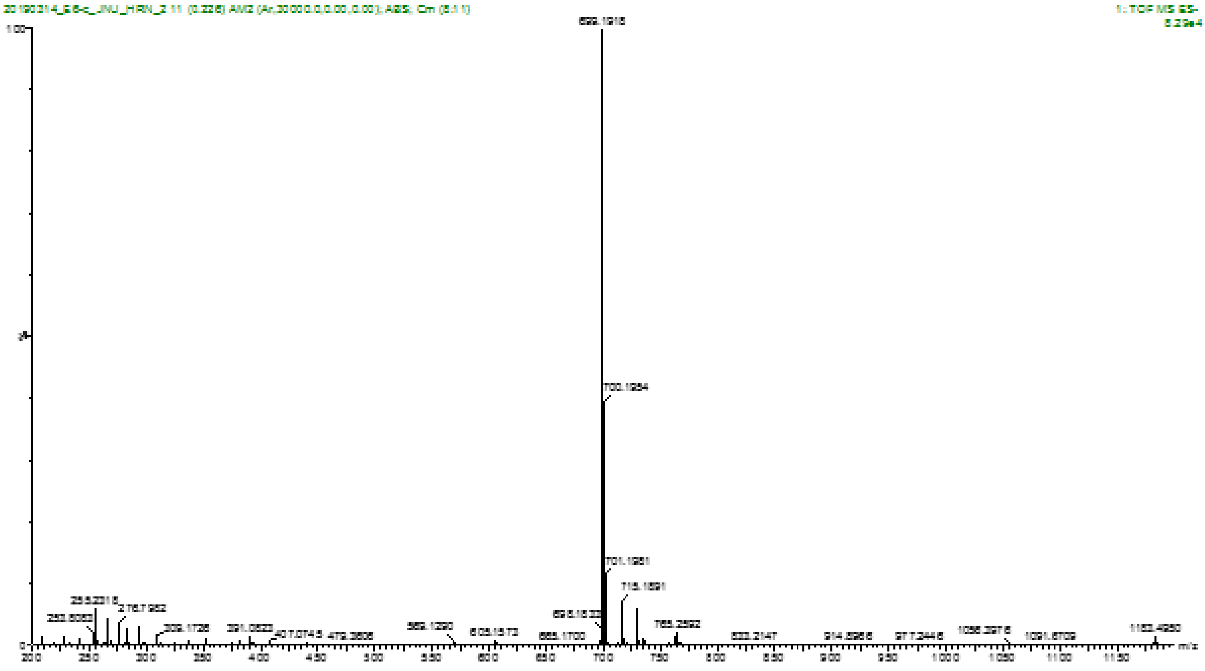

Figure S32. ESI-MS spectrum of compound **4**.

Elemental Composition Report

Single Mass Analysis

Tolerance = 10.0 PPM / DBE: min = -1.5, max = 50.0

Element prediction: Off

Number of isotope peaks used for i-FIT = 3

Monoisotopic Mass, Even Electron Ions

100 formula(e) evaluated with 3 results within limits (all results (up to 1000) for each mass)

Elements Used:

C: 1-50 H: 1-50 O: 1-30

Minimum: -1.5

Maximum: 500.0 10.0 50.0

Mass	Calc. Mass	mDa	PPM	DBE	i-FIT	Norm	Conf(%)	Formula
699.1921	699.1925	-0.4	-0.6	17.5	308.2	0.009	99.11	C34 H35 O16

Figure S33. HRESI-MS data of compound **4**.

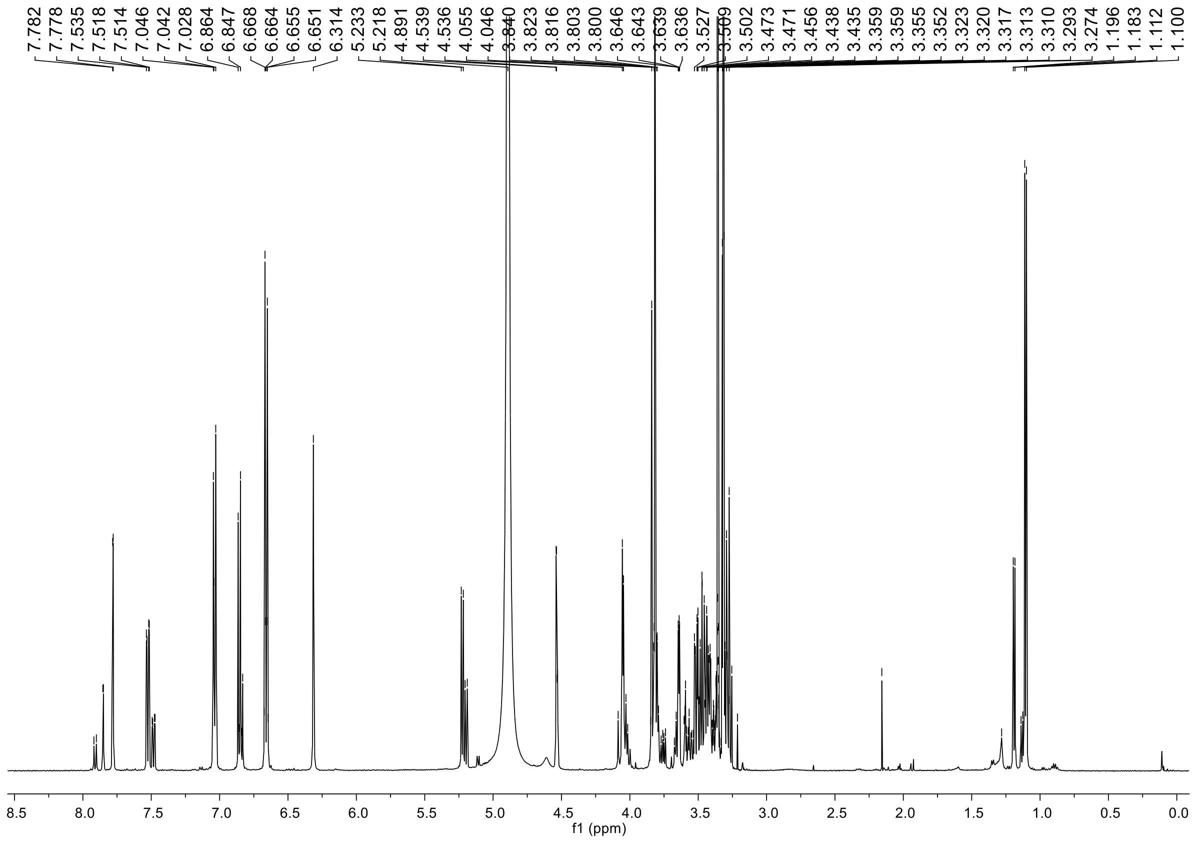

Figure S34. ^{1}H NMR (500 MHz, CD_3OD) spectrum of compounds **5** and **6**.

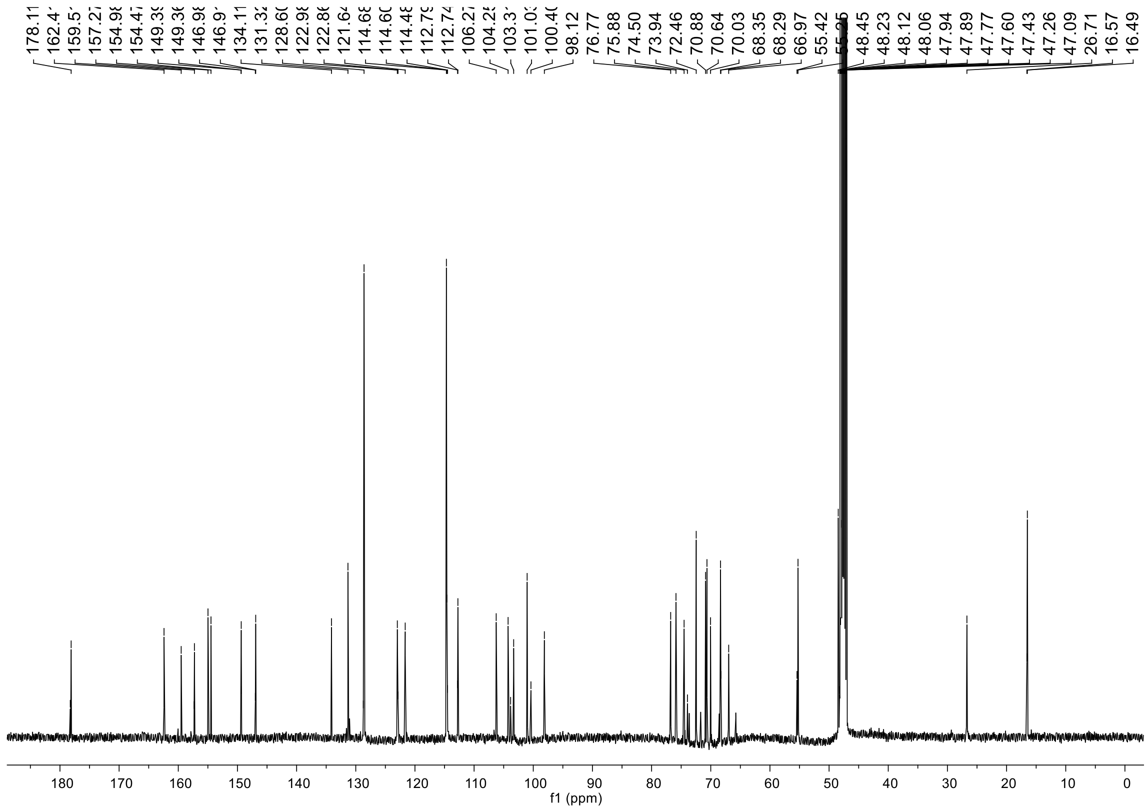

Figure S35. ^{13}C NMR (125 MHz, CD_3OD) spectrum of compounds **5** and **6**.

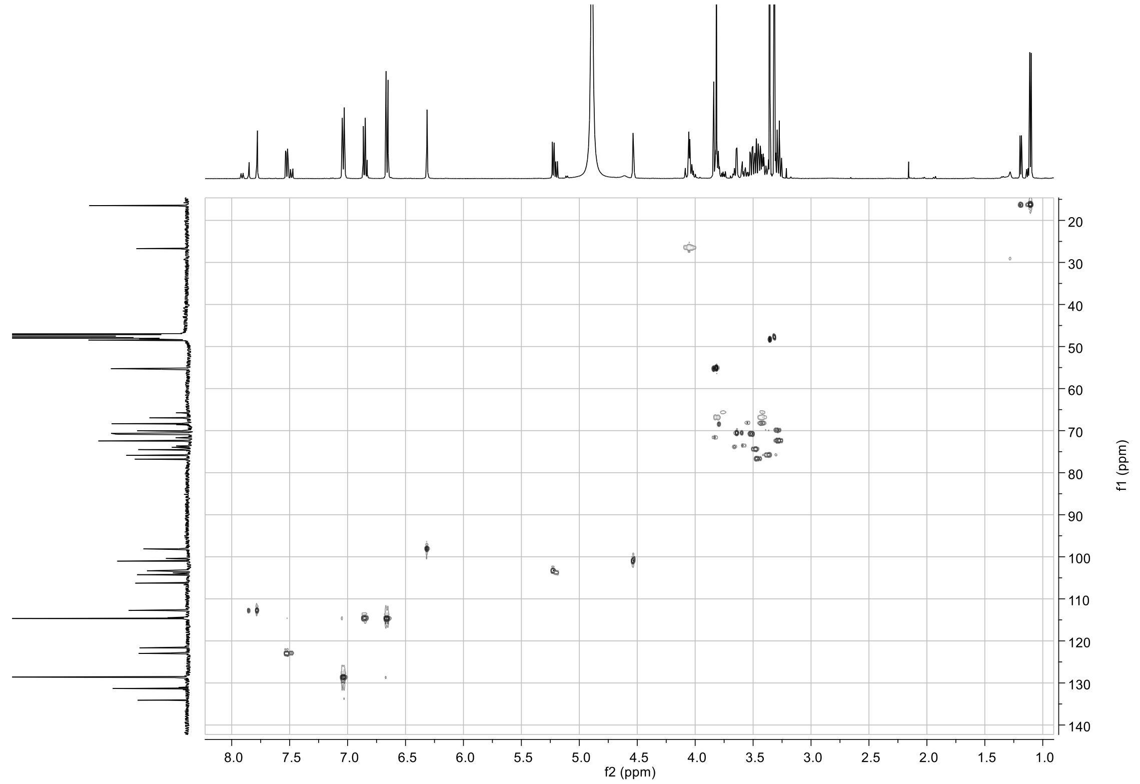

Figure S36. HSQC spectrum of compounds **5** and **6**.

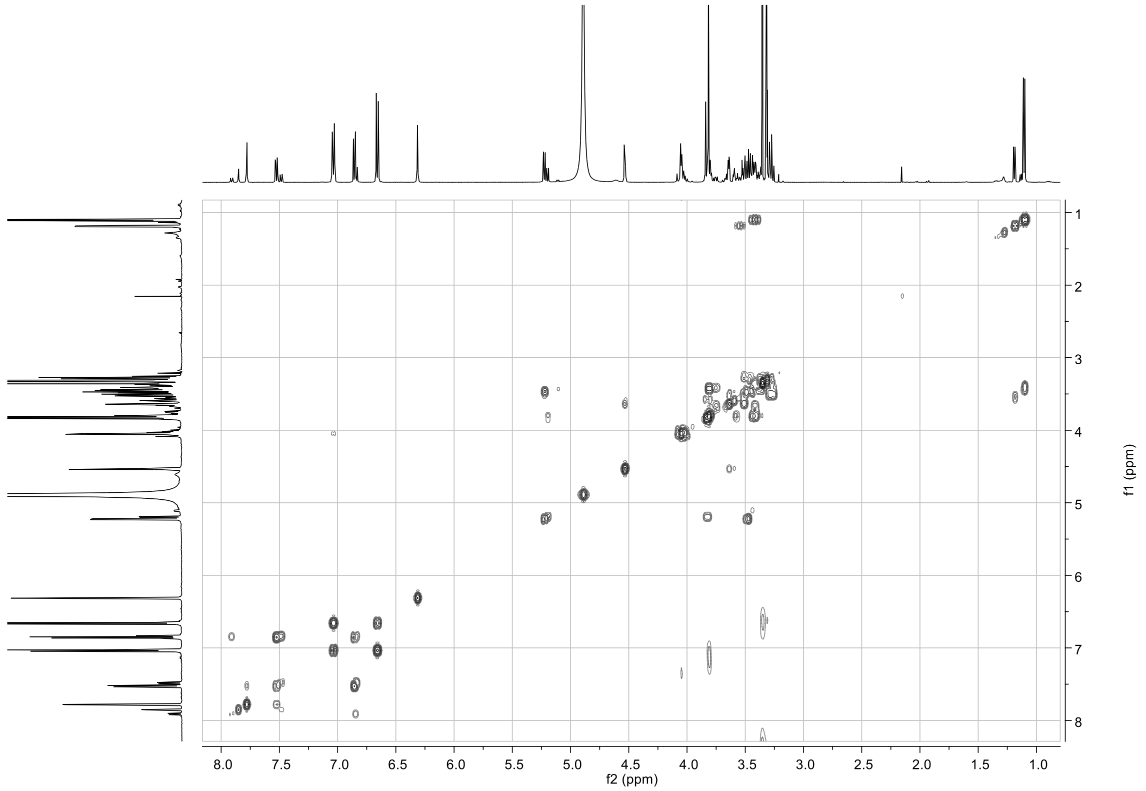

Figure S37. ^{1}H-^{1}H COSY spectrum of compounds **5** and **6**.

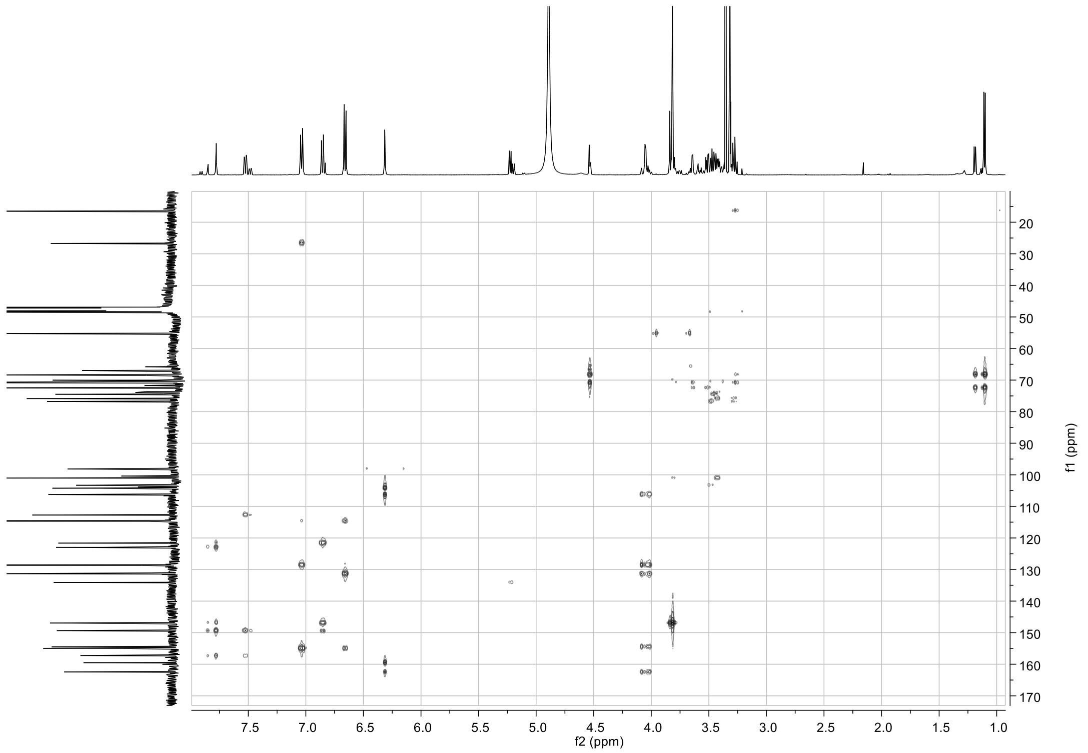

Figure S38. HMBC spectrum of compounds **5** and **6**.

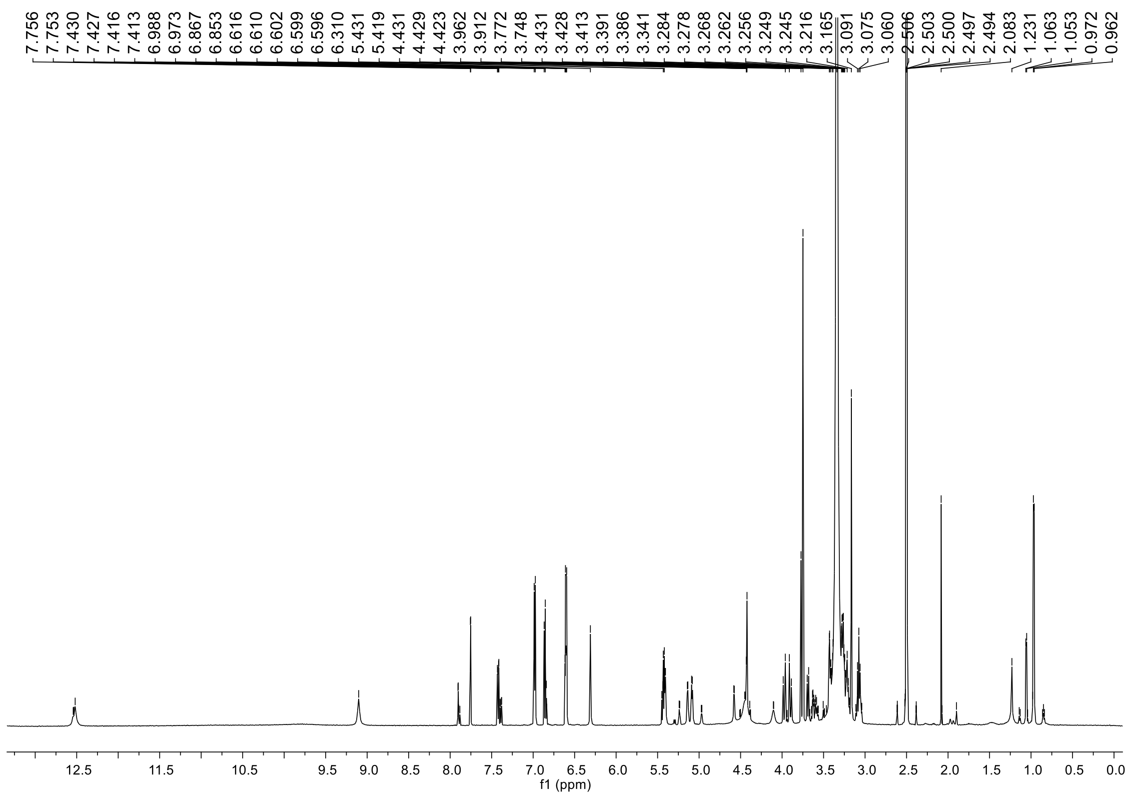

Figure S39. ^{1}H NMR (500 MHz, DMSO-d_6) spectrum of compounds **5** and **6**.

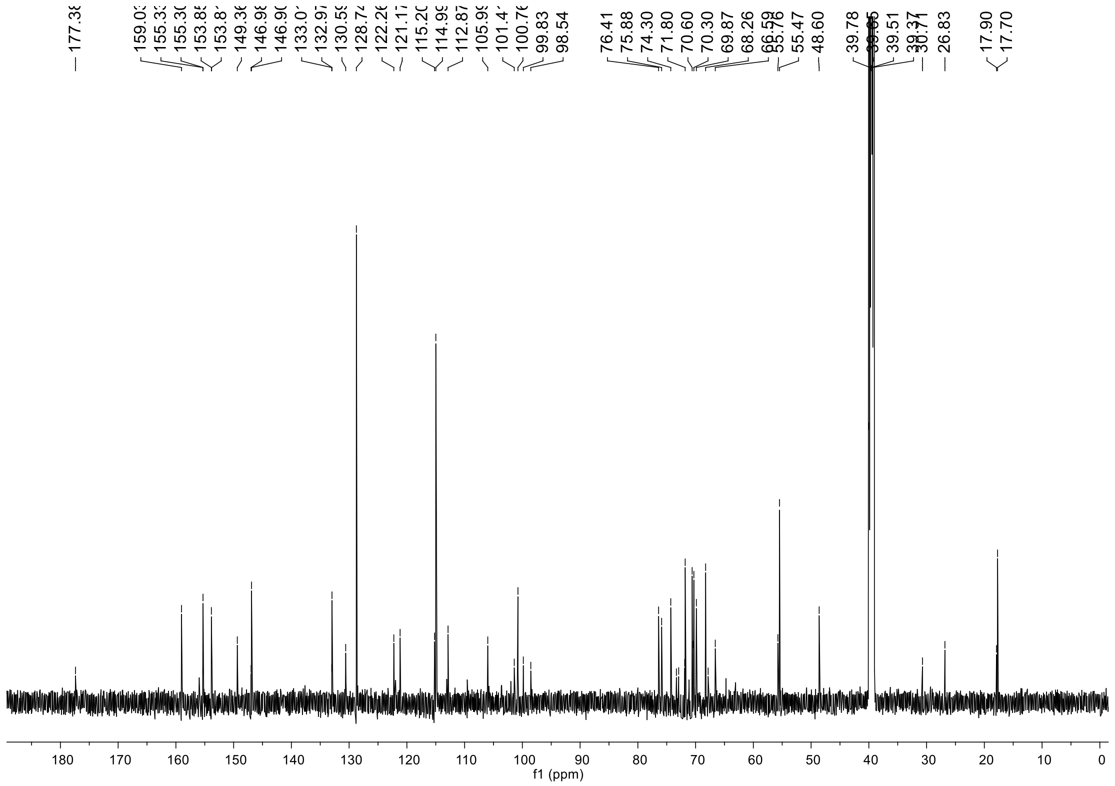

Figure S40. ^{13}C NMR (125 MHz, DMSO-d_6) spectrum of compounds **5** and **6**.

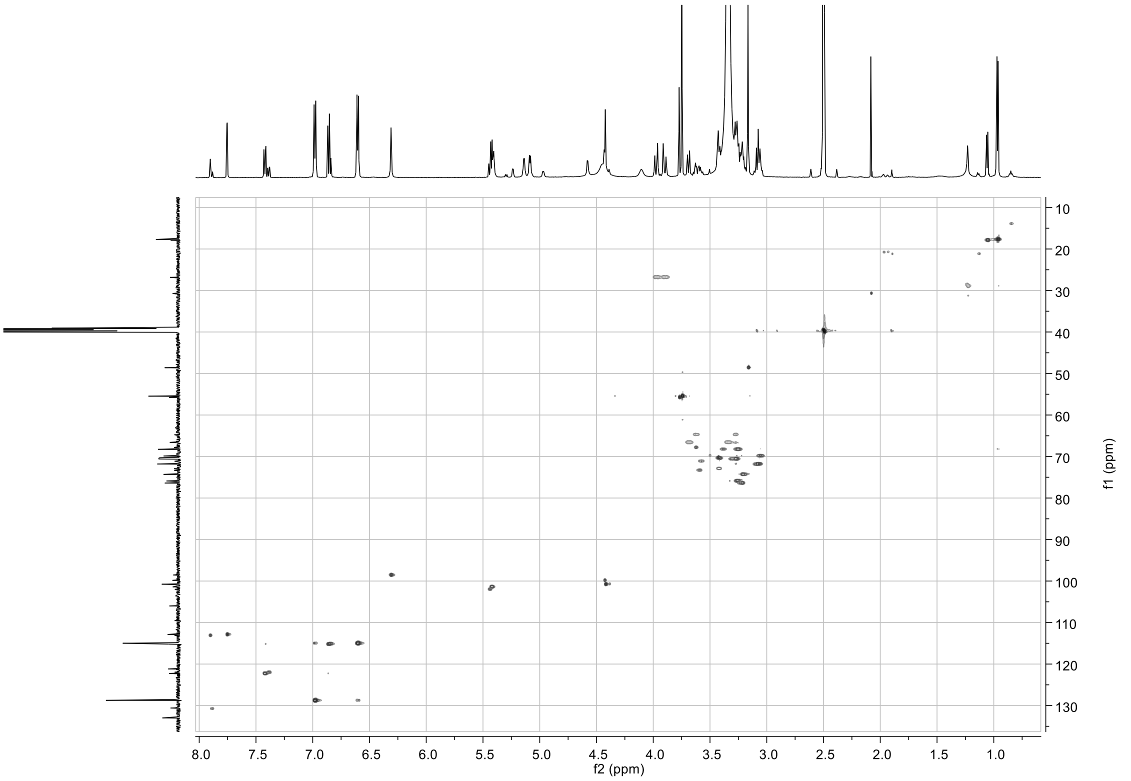

Figure S41. HSQC spectrum of compounds **5** and **6**.

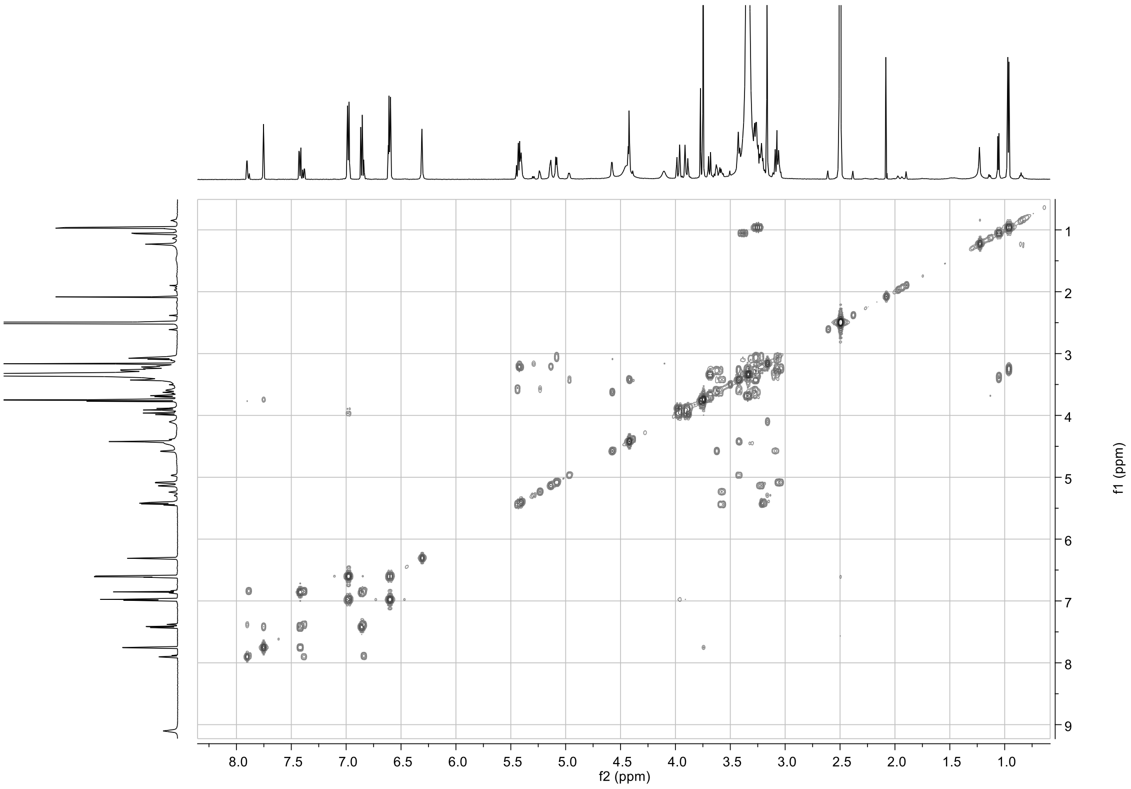

Figure S42. ^{1}H-^{1}H COSY spectrum of compounds **5** and **6**.

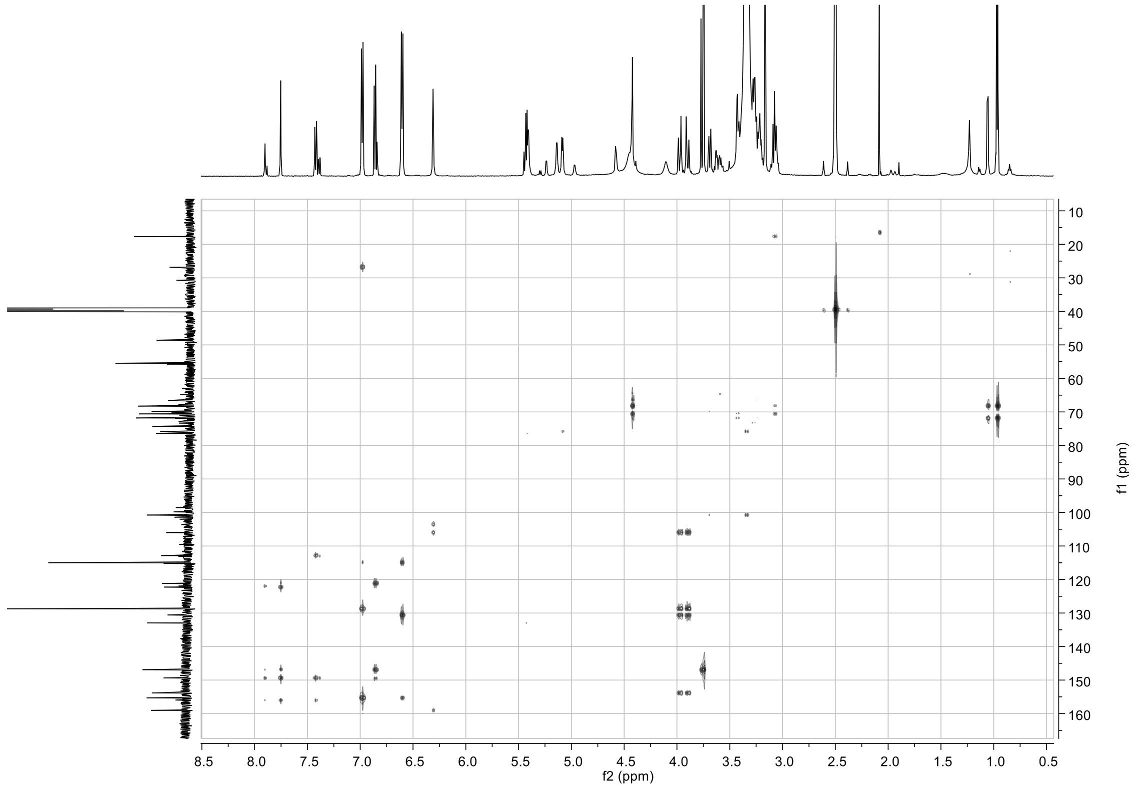

Figure S43. HMBC spectrum of compounds **5** and **6**.

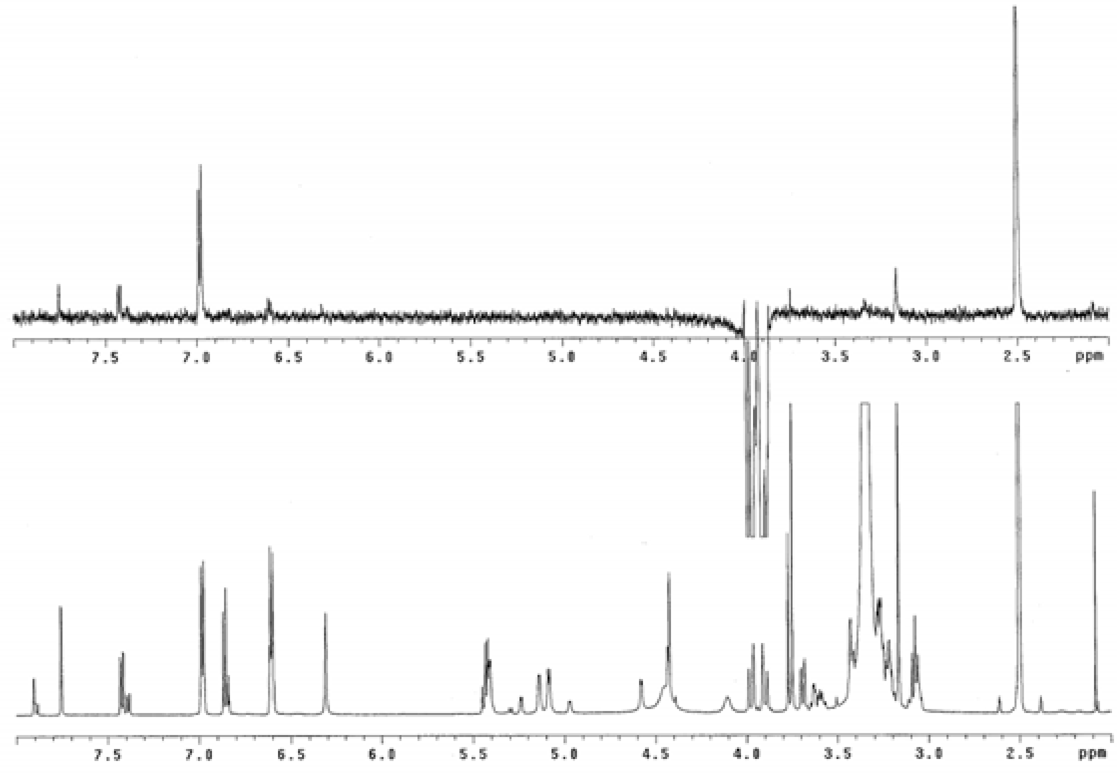

Figure S44. ROESY spectrum of compounds **5** and **6**.

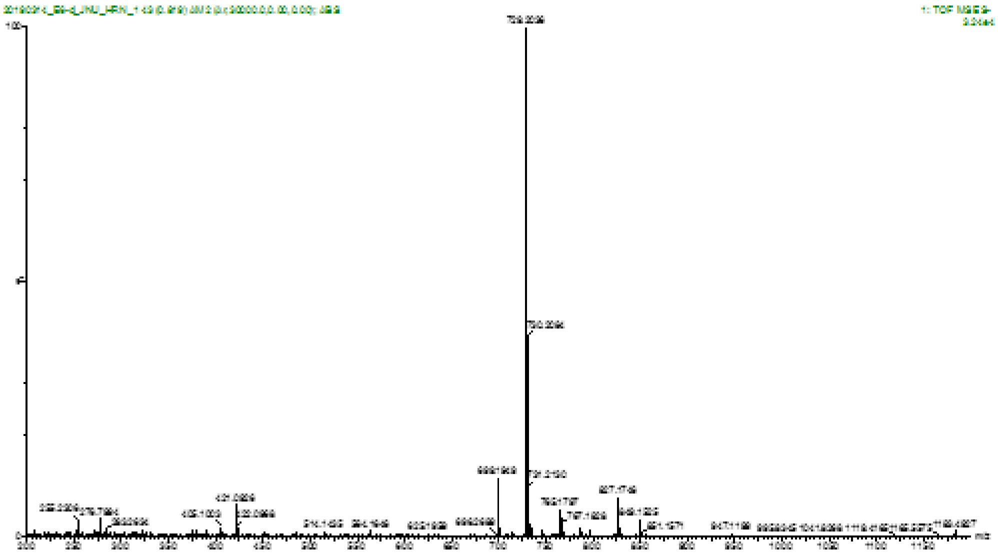

Figure S45. ESI-MS spectrum of compound **5** and **6**.

Elemental Composition Report

Single Mass Analysis

Tolerance = 10.0 PPM / DBE: min = -1.5, max = 50.0

Element prediction: Off

Number of isotope peaks used for i-FIT = 3

Monoisotopic Mass, Even Electron Ions

115 formula(e) evaluated with 2 results within limits (all results (up to 1000) for each mass)

Elements Used:

C: 1-50 H: 1-50 O: 1-30

Minimum:						-1.5		
Maximum:				500.0	10.0	50.0		
Mass	Calc. Mass	mDa	PPM	DBE	i-FIT	Norm	Conf(%)	Formula
729.2036	729.2031	0.5	0.7	17.5	298.8	0.012	98.85	C35 H37 O17

Figure S46. HRESI-MS of compound **5** and **6**.

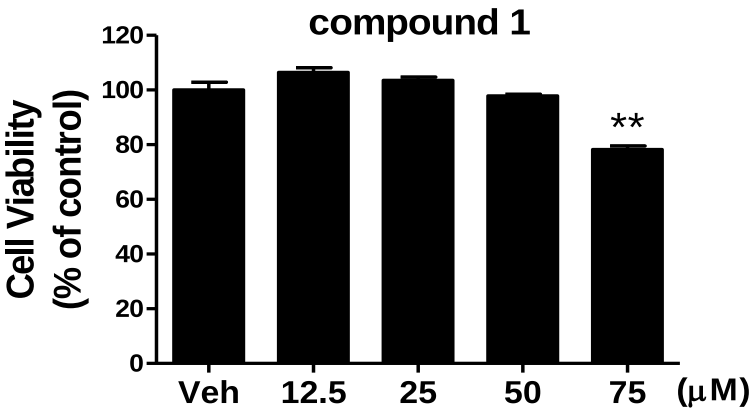

Figure S47. Cell viability of compound **1** in BMDMs.

Cell viability was determined as described in Materials and Methods. Data are presented as means ± SD (n = 3). $^{**}p < 0.01$ compared with the Vehicle (Veh).

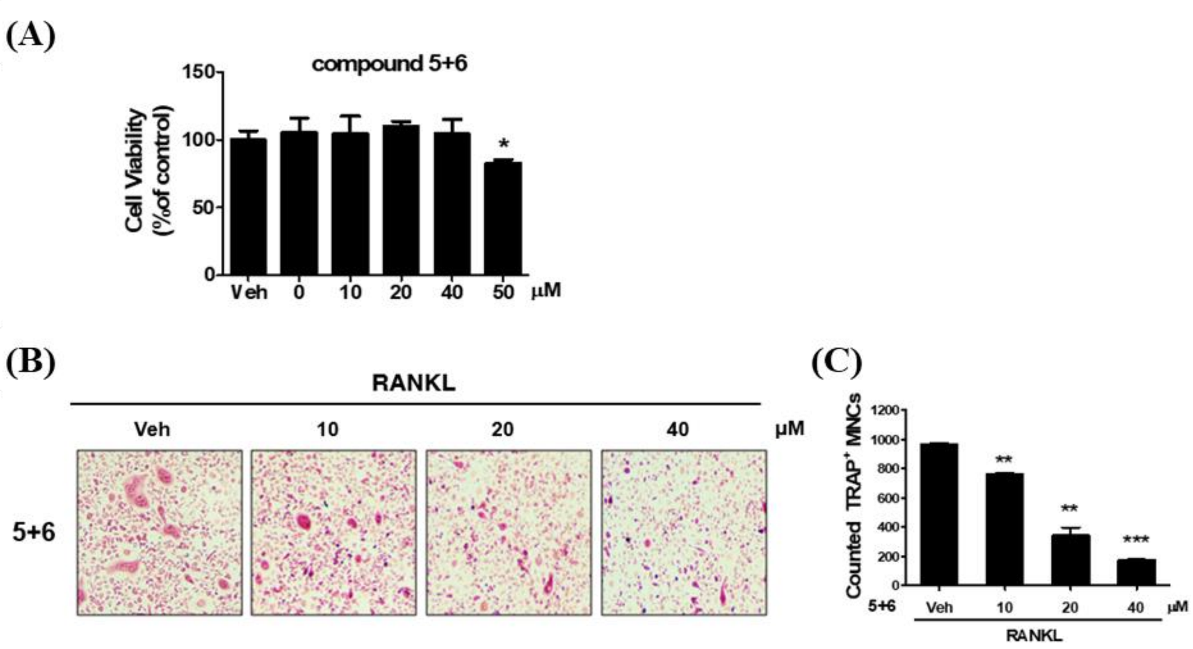

Figure S48. Compound **5**+**6** inhibits osteoclast formation in BMDMs.

(A) Cytotoxicity of Compound **5**+**6** on the BMDMs. BMDMs were cultured on 96-well plate in the presences of M-CSF and treated with the indicated concentration of Compound **5+6**. Cell viability was determined as described in Materials and Methods. Data are presented as means ± SD (n = 3). $^{*}p < 0.05$ compared with the Vehicle (Veh). (B) BMDMs were pretreated with or without **5**+**6** in the presence of M-CSF (25 ng/mL) for 2 h and subsequently stimulated with RANKL (100 ng/mL) for 4 days. Multinucleated cells were visualized by TRAP staining. (C) TRAP-positive multinucleated cells were counted to determine osteoclast numbers. Data are presented as means ± SD (n = 3).
